# Significance of *TP53* Mutational Status-Associated Signature in the Progression and Prognosis of Endometrial Carcinoma

**DOI:** 10.1155/2022/1817339

**Published:** 2022-07-06

**Authors:** Ying Chen, Wancheng Zhao, Fangfang Bi, Xue Pan, Lili Yin, Chengzhi Zhao

**Affiliations:** ^1^Department of Ultrasound, Xiaoshan Traditional Chinese Medical Hospital, Zhouhang, China; ^2^Department of Obstetrics and Gynecology, Shengjing Hospital of China Medical University, Shenyang, China; ^3^Department of Obstetrics and Gynecology, Women and Children's Hospital of Chongqing Medical University, China

## Abstract

**Background:**

TP53 mutations are associated with poor outcome for patients with endometrial carcinoma (EC). However, to date, there have been no studies focused on the construction of TP53 mutational status-associated signature in EC. In this study, we aim to conduct a TP53 mutation-associated prognostic gene signature for EC.

**Methods:**

Hence, we explored the mutational landscape of TP53 in patients with EC based on the simple nucleotide variation data downloaded from The Cancer Genome Atlas (TCGA) database. Differential expression analysis and least absolute shrinkage and selection operator (LASSO)–Cox analysis was used to establish TP53 mutation-associated prognostic gene signature. The overall survival rate between the high-risk and low-risk groups was compared by the Kaplan–Meier (K-M) method.

**Results:**

We found that the TP53 mutation was associated with poor outcome, older age, lower BMI, and higher grade and stage of EC in patients. A TP53 mutational status-associated signature was established based on transcriptome profiling data. Moreover, the patients in TCGA database were categorized into high- and low-risk groups. Kaplan–Meier (K-M) analysis indicated that the patients in the high-risk group have poor survival outcome. Furthermore, receiver operating characteristic (ROC) curves confirmed the robust prognostic prediction efficiency of the TP53 mutational status-associated signature. Finally, the prognostic ability was successfully verified in the other two datasets from cBioPortal database as well as in 60 clinical specimens. Univariate (hazard ratio (HR) = 1.041, 95%CI = 1.031–1.051, *p* < 0.001) and multivariate (hazard ratio (HR) = 1.029, 95%CI = 1.018–1.040, *p* < 0.001) Cox regression analyses indicated that the TP53 mutational status-associated signature could be used as an independent prognostic factor for EC patients.

**Conclusion:**

In summary, our research constructed a powerful TP53 mutational status-associated signature that could be a potential novel prognostic biomarker and therapeutic target for EC.

## 1. Introduction

Endometrial carcinoma (EC), which originates from the endometrial epithelium, is the second most common malignancy of the female reproductive system [1]. Although surgery, chemotherapy, and immunotherapy for EC have led to some improvements in the clinical outcome [2], patient mortality rate is still high [3]. Therefore, it is of practical clinical significance to further explore the pathogenesis of EC at the molecular level and to evaluate and predict the survival rate of patients by studying the prognostic signature of EC [4, 5].

Tumor protein P53 (*TP53*), located on the short arm of chromosome 17 (17p13.1) [6, 7], has a broad spectrum of mutations in human cancers, including allelic loss, deletion, insertion, and point mutations [8]. Chromosomal deletion of *TP53* gene is associated with the occurrence, chemotherapy resistance, and poor prognosis of many tumors [9]. Notably, about 70–80% of mutations in the *TP53* gene are missense mutations caused by the substitution of a single nucleotide, which consequently changes the corresponding amino acid residues. This change, especially the change of arginine residues, can significantly affect *TP53* gene activity [10]. Moreover, the *TP53* protein is inactivated in more than half of tumors [11]. A mutant *TP53* protein not only loses its tumor suppressor function but may also acquire a functional expression similar to an oncogene, promoting the occurrence and development of cancer [12, 13]. Therefore, *TP53* could potentially be a novel biomarker of tumor prognosis and an effective therapeutic target.

Previous studies have confirmed the prognostic value of *TP53* mutations in EC [14–16]. However, so far, no studies have focused on the construction of a *TP53* mutational status-associated signature in EC. Hence, this study is aimed at constructing a TP53 mutational status-associated signature based on The Cancer Genome Atlas (TCGA) database.

## 2. Materials and Methods

### 2.1. Data Acquisition

A dataset with 529 patients with EC including simple nucleotide variation, transcriptome profiling datasets, and clinical data was acquired from TCGA and was utilized as the training dataset (https://portal.gdc.cancer.gov/). The ucec_tcga_pan_can_atlas_2018 and ucec_tcga_pub datasets, which contained the information of 527 and 331 patients with EC, respectively, along with their transcriptome profiling datasets and corresponding clinical information, were downloaded from the cBioPortal database (http://www.cbioportal.org/study/summary?id=ucec_tcga) and used as validation datasets. The deadline for the dataset was June 2020. Inclusion criteria for sample screening included (1) primary endometrial cancer confirmed by pathology without any preoperative radiotherapy or chemotherapy and (2) prognostic information complete without deletion. Finally, this study has been performed according to the REMARK Guidelines. The baseline information of the EC patients is shown in Table 1.

### 2.2. Specimen Collection

A total of 60 patients with EC that were admitted to the Obstetrics and Gynecology Department of the Shengjing Hospital of China Medical University from January 2016 to December 2016 were selected as the research objects. The ages of patients ranged from 26 to 76 years, and the average age was 56.22 ± 10.51 years. In terms of FIGO stage, 26 cases were at stage I, 12 were at stage II, 14 cases were at stage III, and 8 cases were at stage IV. In terms of pathological grades, there were 21, 14, and 25 cases at G1, G2, and G3, respectively. This study was approved by the ethics committee of Shengjing Hospital of China Medical University, and informed consent was obtained from all patients and healthy participants. In addition, all methods were performed in accordance with the relevant guidelines and regulations.

### 2.3. Identification of Differentially Expressed Genes

The “edgeR” package in R was used to screen differentially expressed genes (DEGs) between patients with *TP53* mutation or not. Inclusion criteria was log|FC| > 1 and *p* < 0.05. The cut-off *p* value was 0.029. The “ggplot2” package in R was used to draw the volcano map, and the “ComplexHeatmap” package was used to draw the heat map in order to show the differential expression in patients with *TP53* mutation or not.

### 2.4. Construction and Validation of the *TP53* Mutational Status-Associated Signature

The “Survival” package in R was performed to obtain the DEGs associated with prognostic value according to univariate Cox regression analysis. DEGs with significant prognostic value (*p* < 0.001) were screened to establish the *TP53* mutational status-associated signature using LASSO–multivariate Cox analysis. The risk score for each patient was calculated using the following formula: risk score = *Σ* (regression coefficient × gene expression of DEGs). The median value of risk score was used to classify the patients into high- and low-risk groups, and the K-M and log-rank method was used to compare the overall survival outcome between the two groups. A receiver operating characteristic (ROC) curve was plotted to evaluate the prognostic ability of the *TP53* mutational status-associated signature at different time endpoints using the “Survival” and “timeROC” in R software. In addition, to evaluate the predictive performance of the *TP53* mutational status-associated signature, we verified our analysis results using the ucec_tcga_pan_can_atlas_2018 and ucec_tcga_pub datasets from the cBioPortal database, as well as in 60 clinical specimens.

### 2.5. RT-qPCR Analysis

Trizol was used to extract the total RNA of EC samples (Takara, Japan), and then, complimentary DNA (cDNA) was synthesized using the PrimeScript RT kit (Takara, Japan), following the manufacturer's instructions. The cDNA was amplified using SYBR Premix Ex Taq kit (Takara, Japan), and mRNA hydrolevel was detected using an ABI Prism 7000 fluorescence quantitative PCR assay (Applied Biosystems, Waltham, MA, USA). GAPDH was used as the internal reference, and mRNA expression in the *TP53* mutational status-associated signature was calculated by the 2^-*ΔΔ*CT^ method. The sequences of primers used for RT-qPCR are displayed in Supplementary Table 1. Then, we established a *TP53* mutational status-associated signature as per the method used for the training dataset based on the expression level of mRNAs. The risk scores of 60 clinical specimens were obtained, and specimens were classified into high- and low-risk groups.

### 2.6. Establishment of the Nomogram Model Based on the *TP53*-Associated Signature

To maximize clinical decision-making, the “rms” package in the R software was used to conduct a nomogram based on the expression level of genes in the *TP53* mutational status-associated signature. After the clinicians input the expression values of each gene in the *TP53* mutational status-associated signature for a specific EC patient into the nomogram, the corresponding score values in the score scale were obtained. Then, the resulting score values were added into the total score scale. Finally, a vertical line was drawn on the survival scale to estimate the survival rates at 1, 3, and 5 years. Calibration plots were used to evaluate calibrating ability. The closer the predicted value is to the actual value, the better the nomogram can be corrected. Decision curve analysis (DCA) curves were constructed to evaluate the efficacy of the histogram for prognosis prediction in different sample sets [17].

### 2.7. Statistical Analysis

Univariate and multivariate Cox regression analyses were used to evaluate the independence of *TP53* mutational status-associated signature for EC. The total number of mutations in the DNA of cancer cells (TMB) of the patients with EC in TCGA database was calculated by Perl software. The Wilcoxon rank-sum test was used for comparative analysis between two groups. All statistical analyses were performed using the R language (version 3.6.2). Bilateral test *p* < 0.05 was considered statistically significant.

## 3. Results

### 3.1. *TP53* Mutational Status in EC

**Figure 1:**
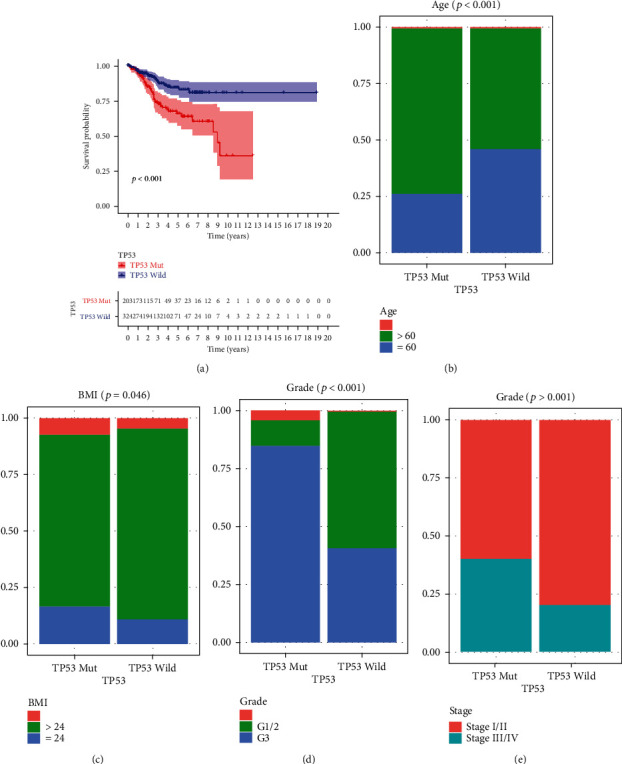
TP53 mutational status in EC patients from TCGA dataset. (a) Kaplan–Meier (K-M) analysis confirmed that the patients with TP53 mutation have poor survival outcome. (b–e) TP53 mutation was related to poor outcome, older age, lower BMI, and higher levels of grade and stage of patients with EC.

Based on the *TP53* mutation data from TCGA dataset, we found that the mutation frequency of the *TP53* was 37%. As expected, K-M analysis confirmed that the patients with *TP53* mutation had poor survival outcome (Figure 1(a), *p* < 0.001). Moreover, Figures 1(b)–1(e) reveal that the *TP53* mutation was related to poor outcome, older age, lower BMI, and higher grade and stage of EC in patients (*p* < 0.05).

### 3.2. Construction of the *TP53* Mutational Status-Associated Signature

**Figure 2:**
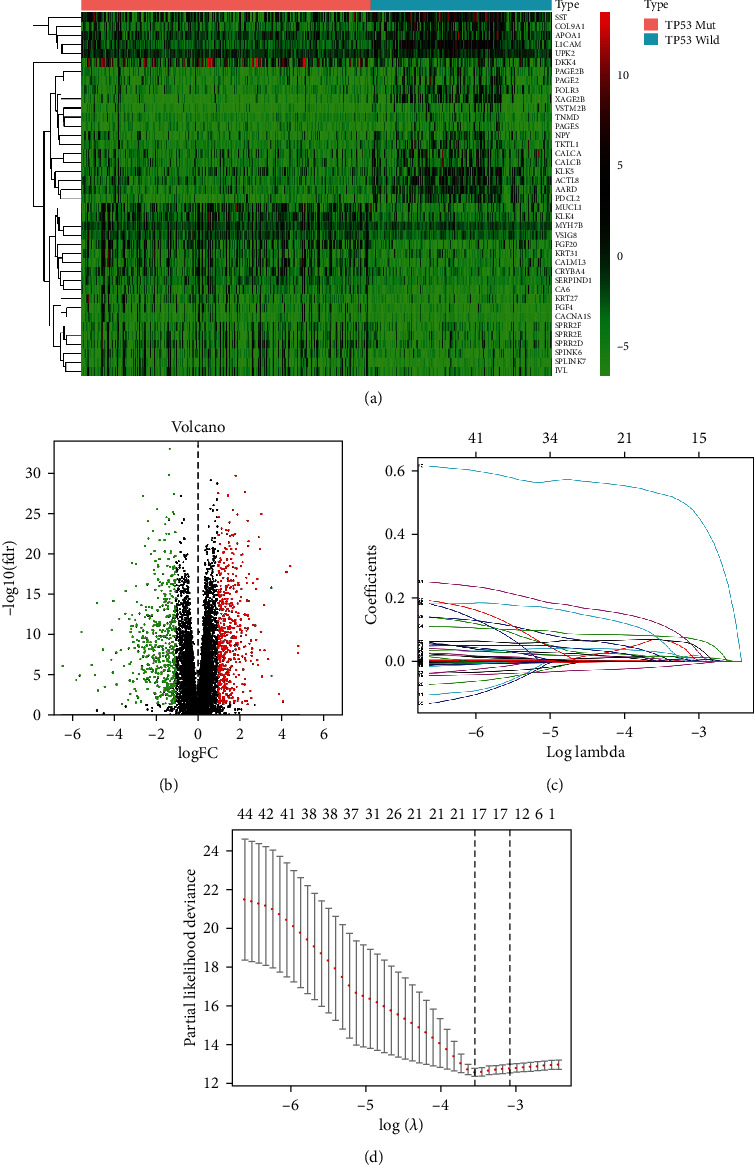
Construction of the TP53 mutational status-associated signature. (a) Heat map showing the top 40 DEGs between patients with TP53 mutation or not. (b) Volcano plot showing the DEGs between patients with TP53 mutation or not. (c) Regression coefficient of 17 DEGs based on the LASSO model. (d) Variables were screened by 10-fold cross-validation method in the LASSO model. When the number of DEG variables was 17, the *λ* value corresponding to the minimum partial likelihood deviation was obtained.

**Figure 3:**
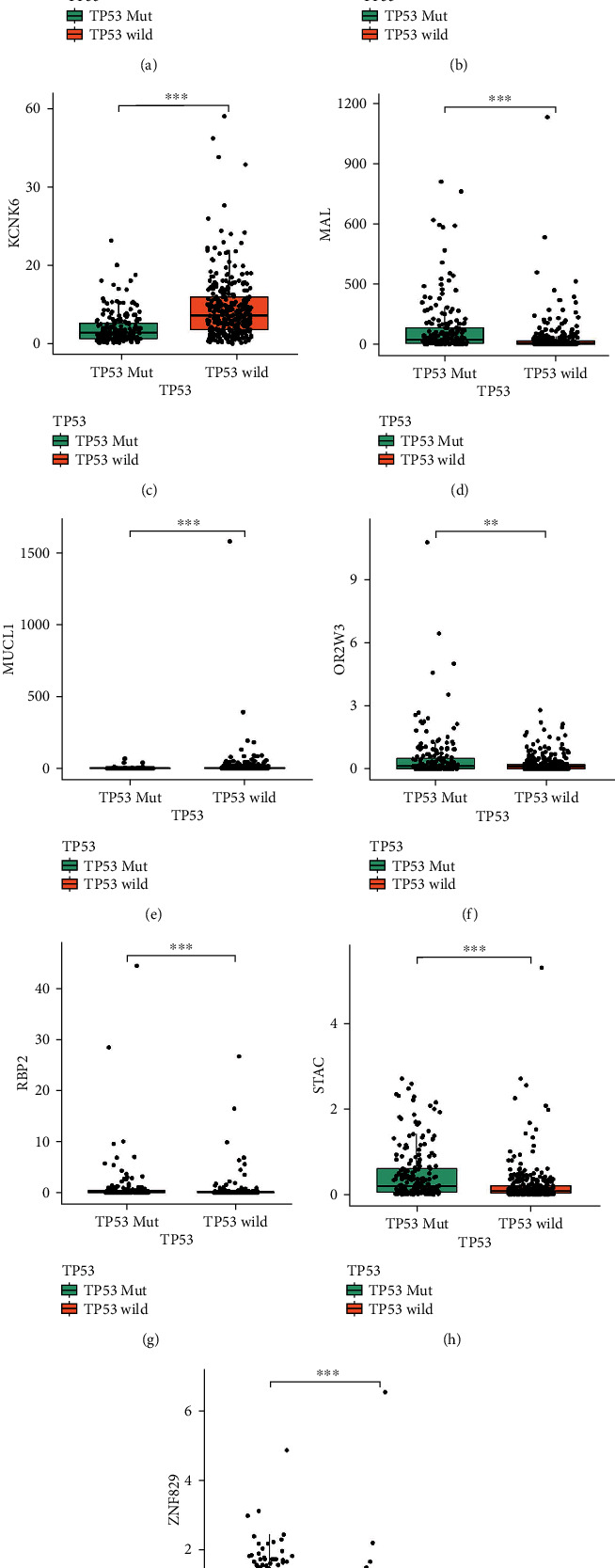
The relative expression level of nine prognostic genes (*ERBB2*, *GLOD5*, *KCNK6*, *MAL*, *MUCL1*, *OR2W3*, *RBP2*, *STAC*, and *ZNF829*) between patients with *TP53* mutation or not: (a) *ERBB2*; (b) *GLOD5*; (c) *KCNK6*; (d) *MAL*; (e) *MUCL1*; (f) *OR2W3*; (g) *RBP2*; (h) *STAC*; (i) *ZNF829*.

A total of 1058 DEGs were identified based on *p* < 0.05 and log_2_|*FC*| > 1 screening standard (Supplementary Table 2; Figures 2(a) and 2(b)). Univariate Cox regression preliminarily screened 50 DEGs associated with the prognosis of patients with EC (Supplementary Table 3, *p* < 0.001), and then, LASSO regression analysis identified 17 key DEGs (Figures 2(c) and 2(d)). Of these, nine were further screened by forward stepwise method, and then, a *TP53* mutational status-associated signature was constructed. Moreover, multivariate Cox regression analysis was used to calculate the risk score of each EC patient, with the following formula: risk score = 0.0012 × exp ERBB2 + 0.1121 × exp GLOD5 − 0.0555 × exp KCNK6 + 0.0024 × exp MAL + 0.0029 × exp MUCL1 + 0.2903 × exp OR2W3 + 0.0471 × exp RBP2 + 0.7545 × exp STAC + 0.3902 × exp ZNF829 (Table 2). The difference analysis between *TP53* mutation and *TP53* wild-type group revealed that ERBB2, GLOD5, KCNK6, MAL, OR2W3, STAC, and ZNF829 were expressed at higher levels in the *TP53* mutation group, while MUCL1 and RBP2 were expressed at higher levels in the *TP53* wild-type group (Figures 3(a)–3(i)).

### 3.3. Evaluation of the *TP53* Mutational Status-Associated Signature

**Figure 4:**
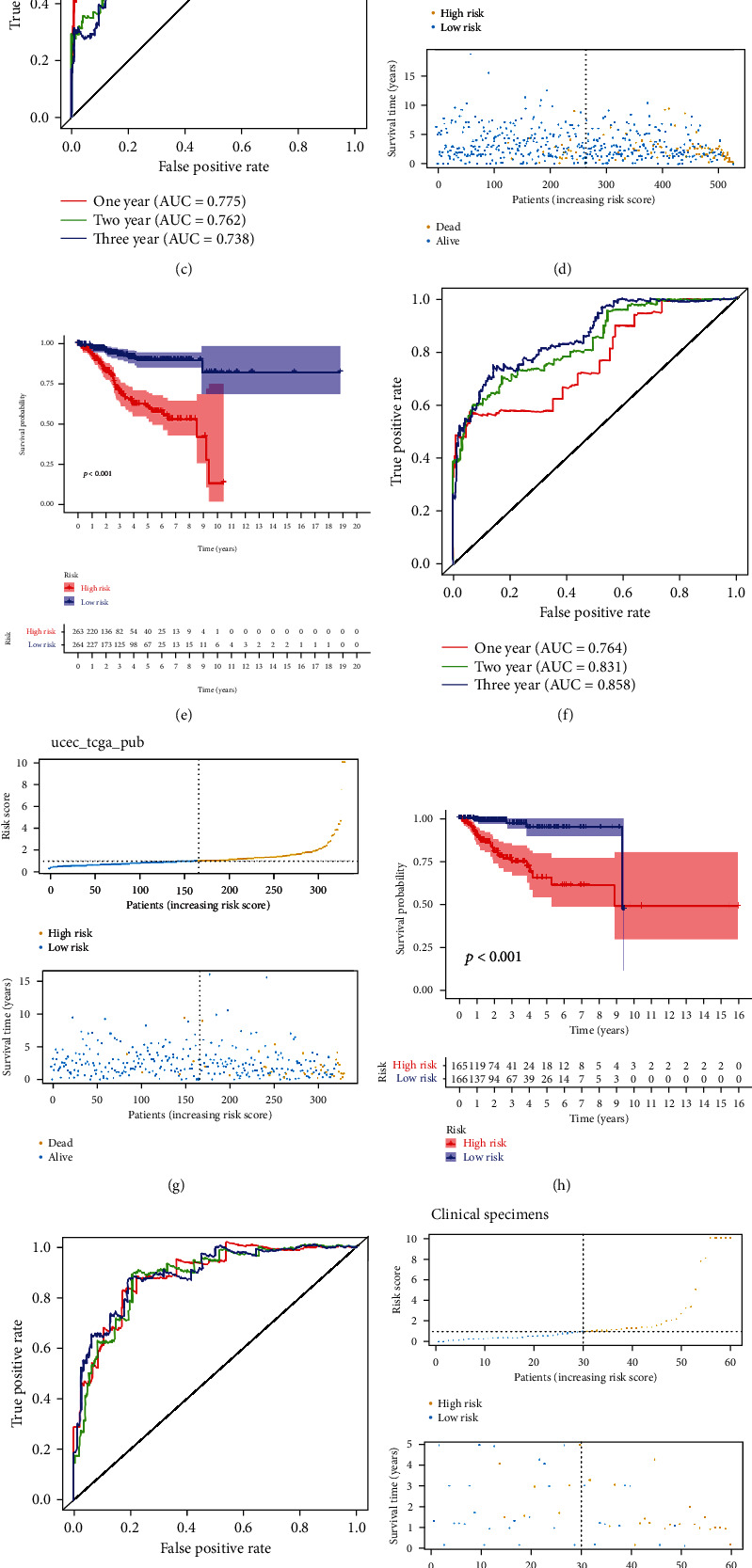
Evaluation of the *TP53* mutational status-associated signature in training and validation datasets, as well as in clinical specimens. (a–c) Risk score, survival status, K–M curve, and ROC curve in TCGA; (d–f) ucec_tcga_pan_can_atlas_2018; (g–i) ucec_tcga_pub dataset; (j–l) clinical specimens.

We then evaluated and validated the prognostic efficacy of the *TP53* mutational status-associated signature in both training and validation datasets. The risk scores and survival status of each patient with EC have been determined (Figures 4(a), 4(d), 4(g), and 4(j)). K-M analysis showed that patients in the low-risk group have longer survival time than those in the high-risk group (Figures 4(b), 4(e), 4(h), and 4(k), *p* < 0.001). Moreover, ROC analysis showed that the overall survival rates of EC patients at 1, 3, and 5 years in TCGA dataset were 0.775, 0.762, and 0.738, respectively (Figure 4(c)). In the ucec_tcga_pan_can_atlas_2018 dataset, the overall survival rates at 1, 3, and 5 years were 0.764, 0.831, and 0.858, respectively (Figure 4(f)), whereas in the ucec_tcga_pub dataset, the overall survival rates at 1, 3, and 5 years were 0.886, 0.878, and 0.890, respectively (Figure 4(i)).  Figure 4(l) reveals that the 1-, 3-, and 5-year overall survival rates (AUC) in clinical specimens were 0.925, 0.851, and 0.826, respectively.

### 3.4. Independent Prognostic Value of the *TP53* Mutational Status-Associated Signature

**Figure 5:**
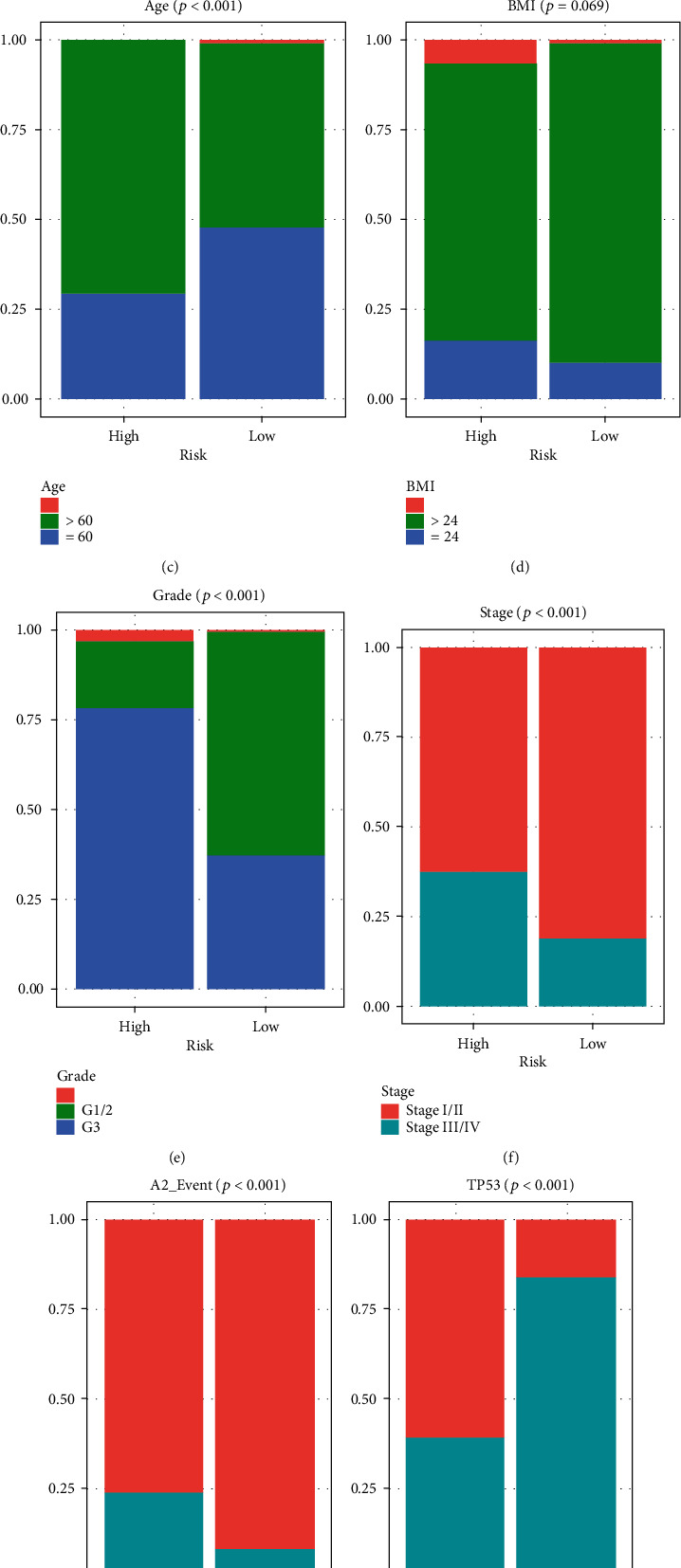
Independent prognostic value of the *TP53* mutational status-associated signature. (a) Univariate Cox regression analyses. (b) Multivariate Cox regression analyses. (c–h) Patients with older age, higher EC grade and stage, dead event, and TP53 Mut were more distributed in the high-risk group.

With overall survival as the dependent variable, the risk score is calculated by the *TP53* mutational status-associated signature, age, BMI, pathological stage, and grade in TCGA dataset. Univariate (hazard ratio (HR) = 1.041, 95%CI = 1.031–1.051, *p* < 0.001) and multivariate (hazard ratio (HR) = 1.029, 95%CI = 1.018–1.040, *p* < 0.001) Cox regression analyses indicate that the *TP53* mutational status-associated signature has significant prognostic value, which could be used as an independent prognostic factor for EC patients (Figures 5(a) and 5(b)). We then investigated correlations between mutational status and the new risk score and clinicopathological variables in TCGA dataset and found that patients with older age, higher EC grade and stage, dead event, and TP53 Mut were more distributed in the high-risk group (Figures 5(c)–5(h), Table 3, *p* < 0.001).

### 3.5. Establishment of the Nomogram Model Based on the *TP53*-Associated Signature

**Figure 6:**
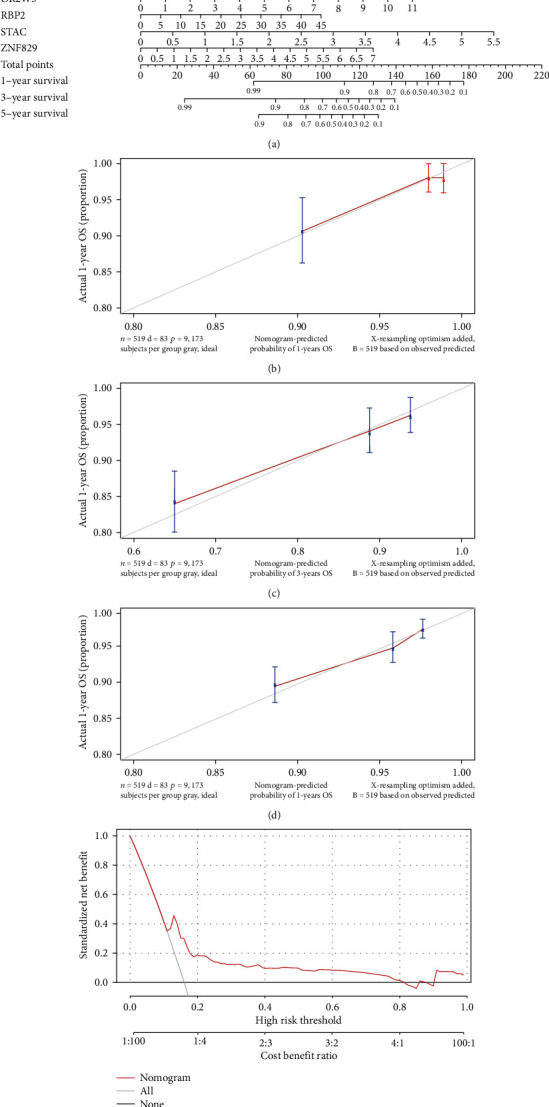
Construction of a nomogram model. (a) A nomogram model predicting survival in EC patients at 1, 3, and 5 years. (b–d) The calibration curve at 1, 3, and 5 years. (e) Decision curve analysis (DCA) curve evaluated the clinical benefit of the nomogram model.

We successfully constructed a nomogram based on the expression levels of the above nine DEGs. After the clinicians input the expression values of nine genes for a specific EC patient into the nomogram, the corresponding score values in the score scale were obtained, and the resulting score values were added into the total score scale. Finally, a vertical line was drawn on the survival scale to estimate the survival rates at 1, 3, and 5 years (Figure 6(a)). Calibration curves showed that the predicted survival rates of patients with EC were in good agreement with the actual survival rates at 1, 3, and 5 years (Figures 6(b)–6(d)). Moreover, DCA results showed that the nomogram had high net income (Figure 6(e)).

### 3.6. Mutational Landscape Associated with the *TP53* Mutational Status-Associated Signature

**Figure 7:**
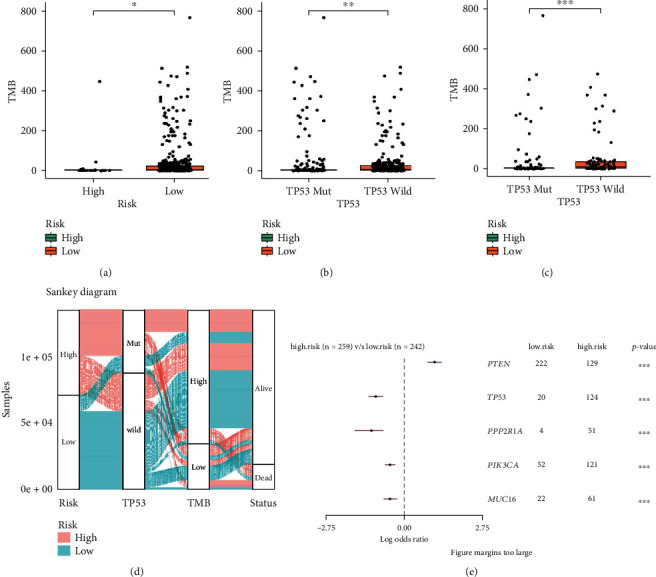
Mutational landscape associated with the *TP53* mutational status-associated signature. (a) Differences in TMB between the high- and low-risk groups. (b) Differences in TMB between EC patients with *TP53* mutation or not in the high-risk group. (c) Differences in TMB between EC patients with *TP53* mutation or not in the low-risk group. (d) Sankey diagram showed the relationship between risk score, *TP53* mutational status, TMB, and survival status. (e) Mutational landscape associated with the *TP53*-associated signature.

TMB is defined as the number of tumor-specific mutations per million coding region bases [18]. Figures 7(a)–7(c) reveal that the patients with *TP53* wild type and those in the low-risk group had higher TMB values. Moreover, the Sankey diagram showed the relationship between risk score, *TP53* mutational status, TMB, and survival status (Figure 7(d)). Finally, we investigated the mutational landscape associated with the *TP53* mutational status-associated prognostic signature and found that *PTEN* had higher mutation frequency in the high-risk group, while *TP53*, *PPP2R1A*, *PIK3CA*, and *MUC16* had low mutation frequencies (Figure 7(e)).

## 4. Discussion

EC is the most common type of cancer in the female reproductive system [19]. In recent years, the understanding of EC has deepened, and some achievements have been made in the treatment and prognostic assessment of EC. However, there has still not been a breakthrough in treatment strategies, and individualized treatment of EC still faces great challenges. Previous studies have reported that the *TP53* mutation is associated with poor outcome of patients with EC, which was confirmed in our research [20, 21]. However, to date, there are still no relevant studies on the development of a *TP53* mutational status-associated signature. In our study, a *TP53* mutational status-associated signature with powerful predictive potential in TCGA dataset was constructed and verified its potential using two datasets from the cBioPortal database, as well as in 60 clinical specimens, indicating that this could be a novel prognostic biomarker and therapeutic target for EC.

This *TP53* mutational status-associated signature was constructed using LASSO–Cox analyses of identified key DEGs, which included *ERBB2*, *GLOD5*, *KCNK6*, *MAL*, *MUCL1*, *OR2W3*, *RBP2*, *STAC*, and *ZNF829*. To explore how these genes are involved in the development of EC, we reviewed the previous studies.

Erb-B2 receptor tyrosine kinase 2 (*ERBB2*), also known as *HER2*, is a member of the ERBB family [22]. *ERBB2*, as a proto-oncogene, has been confirmed to be upregulated in EC tissues and is related to poor prognosis [23]. Several targeted therapies for *ERBB2*, such as trastuzumab, pertuzumab, and lapatinib, have been used in the clinical setting [24]. Potassium channel subfamily K member 6 (KCNK6) is the background potassium channel belonging to the potassium channel family of double pore domain. KCNK6 is upregulated in thyroid carcinoma and breast cancer and is related to the proliferation, invasion, and migration of breast tumor cells [25, 26]. Myelin and lymphocyte protein (*MAL*) encodes T lymphocyte maturation-related proteins and plays a role in T cell differentiation. Downregulated *MAL*, as a tumor suppressor gene, was associated with a variety of human epithelial malignancies [27]. A study revealed that the *MAL* can be used for the early diagnosis of EC [28]. Mucin-like 1 (*MUCL1*), also known as *SBEM*, is a breast-specific gene that is associated with the occurrence, progression, prognosis, and chemotherapy response of breast cancer [29]. *OR2W3*, which belongs to the *ORS* gene family, has been revealed to be related to the progression of breast cancer [30]. Retinoblastoma-binding protein 2 (RBP2) belongs to the JARID protein family and is responsible for histone demethylase (HDM) activity. As a chromatin-modifying enzyme, it has been shown to be involved in the development and progression of a variety of cancers [31]. Src homology three (*SH3*) and cysteine-rich domain (*STAC*) encodes a cysteine-rich protein containing the SH3 domain, which is mainly expressed in neurons and may be involved in neuron-specific signal transduction [32]. So far, no relevant studies have been found on the *GLOD5* and *ZNF829* genes. Although most of these genes have not been previously reported in EC, they have been found to play an important role in the development of other tumors [30, 33–35].

To evaluate and validate the prognostic value of the *TP53* mutational status-associated signature in both the training and validation datasets, as well as in clinical specimens, an ROC curve at 1, 3, and 5 years was plotted. We found that the mean of AUC value was more than 0.80, indicating that the TP53 mutational status-associated signature has a powerful prognostic ability.

## 5. Conclusion

In summary, we conducted and validated a *TP53* mutational status-associated signature with robust predictive potential. To our knowledge, this is the first study to do so. The *TP53* mutational status-associated signature could potentially be used as a novel prognostic biomarker and therapeutic target for EC.

## Figures and Tables

**Table 1:** The baseline information of the EC patients.

Characteristic	TCGA (529)	ucec_tcga_pan_can_atlas_2018 (527)	ucec_tcga_pub (331)	Clinical specimens (60)
Age (years)				
≤60	202	204	119	20
>60	324	321	212	40
None	3	2	0	0
BMI				
≤24	69	65	59	11
>24	430	433	261	49
None	30	29	11	0
Grade				
G1/2	215	215	169	35
G3	305	301	155	25
None	9	11	7	0
Stage				
I/II	381	377	239	38
III/IV	148	150	92	22
None		0	0	0
TP53				
Mut	204	192	103	24
Wild	325	335	228	36
None	0	0	0	0
Event				
Alive	444	441	280	33
Dead	83	84	51	27
None	2	2	0	0

**Table 2:** Regression coefficients of the nine *TP53* mutational status-associated prognostic genes.

id	coef	HR	HR.95L	HR.95H	*p* value
ERBB2	0.001239	1.001239	1.000481	1.001998	0.00135
GLOD5	0.112093	1.118617	1.058151	1.182539	7.70*E* − 05
KCNK6	-0.05554	0.945978	0.899089	0.995311	0.032261
MAL	0.002416	1.002419	1.001031	1.003809	0.000632
MUCL1	0.002946	1.002951	1.001758	1.004145	1.22*E* − 06
OR2W3	0.290267	1.336784	1.132684	1.577661	0.000595
RBP2	0.047118	1.048245	0.998892	1.100037	0.055503
STAC	0.754457	2.126455	1.56286	2.893294	1.57*E* − 06
ZNF829	0.390186	1.477256	1.112411	1.961761	0.007017

**Table 3:** Differences in TP53 mutational status and clinicopathological variables according to the risk status in TCGA dataset.

Characteristic	High risk	Low risk	*χ* ^2^	*p*
Age (years)				
≤60	76	124	21.09	*p* < 0.0001
>60	183	134		
None	0	2		
BMI				
≤24	42	26	5.34	0.069
>24	200	221		
None	17	13		
Grade				
G1/2	48	162	104.78	*p* < 0.0001
G3	203	97		
None	8	1		
Stage				
I/II	162	211	22.22	*p* < 0.0001
III/IV	97	49		
None	0	0		
TP53				
Mut	157	42	108.51	*p* < 0.0001
Wild	102	218		
None	0	0		
Event				
Alive	197	239	24.29	*p* < 0.0001
Dead	62	21		
None	0	0		

## Data Availability

All data generated or analyzed during this study are included in this published article.

## Abbreviations

EC: Endometrial carcinoma
TCGA: The Cancer Genome Atlas
LASSO: Least absolute shrinkage and selection operator
K-M: Kaplan–Meier
ROC: Receiver operating characteristic
ERBB2: Erb-B2 receptor tyrosine kinase 2
MAL: Myelin and lymphocyte protein
MUCL1: Mucin-like 1
RBP2: Retinoblastoma-binding protein 2.

## Supplementary Materials

Supplementary 1 Supplementary Table 1: the sequences of primers used for RT-qPCR.

Supplementary 2 Supplementary Table 2: a total of 1058 differentially expressed genes (DEGs), including 525 upregulated genes and 533 downregulated genes, were identified based on *p* < 0.05 and log_2_|*FC*| > 1 screening standard.

Supplementary 3 Supplementary Table 3: univariate Cox regression preliminarily screened 50 DEGs with the prognosis value.
