# Supplementary material for: Significance of TP53 Mutational Status-Associated Signature in the Progression and Prognosis of Endometrial Carcinoma
Source: Oxid Med Cell Longev. 2022 Jul 6;2022:1817339. doi: 10.1155/2022/1817339 (PMC9280614; doi:10.1155/2022/1817339)
Supplement: Supplementary 1 — Supplementary Table 1: the sequences of primers used for RT-qPCR. [file 1817339.f1.docx]

| Gene | Sequences of the primers |
| --- | --- |
| ERBB2 | F:5' CGCAGGCAGTGATGAGAGTGAC 3'  R:5’ TGGTGGGCAGGTAGGTGAGTTC3’ |
| GLOD5 | F:5' CCAGGCTGCCAGTCAAGATGTG 3’  R:5’ CGGCTTTGGGTTCAAATTCCTTTCC 3’ |
| KCNK6 | F:5' TTAATGGAGGAGGTGAGGTGTTGAAAG 3’  R:5’ AGGCTGCTGAGGAAGAGTCTATCG 3’ |
| MAL | F:5' ATGGAAGTCTTCATAAAGCCGCAGTAG 3’  R:5’ GACAGCACAGCAGTTGGAGGTTAG 3’ |
| MUCL1 | F:5’ TCACCACCATGAAGTTCTTAGCAGTC 3’  R:5' TCTCAGGGACACACTCTACCATTCG 3’ |
| OR2W3 | F:5’ CTCGTAGGCAACACCACCATCATC 3’  R:5' CCCAGACCCAGGAACAGGAAGAG 3’ |
| RBP2 | F:5' AGAATGGAACCTGGGAGATGGAGAG3'  R:5’ TTGAACACTTGACGGCACACCTG 3’ |
| STAC | F:5’ GGCAGCAGCAGTAGGACTATAAACC 3’  R:5’ GGACACTCTGGACAGCACAATCAC 3’ |
| ZNF829 | F:5' TCAGCCCACATTTCTTATTCCACCTC 3'  R:5’ GCCTTGCCACATTCCTTACATTCATAG 3’ |
| GAPDH | F:5' CAAGGCTGTGGGCAAGGTCATC 3'  R:5’ GTGTCGCTGTTGAAGTCAGAGGAG 3’ |

**Supplementary Table 1** The sequences of primers used for RT-qPCR.
