# Supplementary material for: Significance of TP53 Mutational Status-Associated Signature in the Progression and Prognosis of Endometrial Carcinoma
Source: Oxid Med Cell Longev. 2022 Jul 6;2022:1817339. doi: 10.1155/2022/1817339 (PMC9280614; doi:10.1155/2022/1817339)
Supplement: Supplementary 2 — Supplementary Table 2: a total of 1058 differentially expressed genes (DEGs), including 525 upregulated genes and 533 downregulated genes, were identified based on p < 0.05 and log2|FC| > 1 screening standard. [file 1817339.f2.docx]

| gene | conMean | treatMean | logFC | pValue | fdr |
| --- | --- | --- | --- | --- | --- |
| DKK4 | 281.2918 | 3.359485 | -6.38768 | 1.77E-07 | 7.98E-07 |
| FGF4 | 2.363284 | 0.044591 | -5.7279 | 1.42E-05 | 4.38E-05 |
| KRT31 | 7.576548 | 0.158833 | -5.57596 | 3.29E-06 | 1.15E-05 |
| CALML3 | 10.29532 | 0.229516 | -5.48725 | 4.86E-12 | 5.36E-11 |
| CACNA1S | 0.498011 | 0.015593 | -4.99718 | 1.23E-07 | 5.72E-07 |
| KRT27 | 3.137647 | 0.108609 | -4.85247 | 2.95E-06 | 1.04E-05 |
| SERPIND1 | 2.225675 | 0.083468 | -4.73687 | 5.49E-16 | 1.38E-14 |
| SPRR2E | 4.05121 | 0.155839 | -4.70023 | 0.000349 | 0.000818 |
| SPINK7 | 1.740915 | 0.078836 | -4.46485 | 1.09E-09 | 7.43E-09 |
| SPRR2D | 8.83407 | 0.424895 | -4.3779 | 0.00011 | 0.000286 |
| IVL | 3.134238 | 0.186464 | -4.07115 | 2.69E-08 | 1.41E-07 |
| KLK4 | 5.914566 | 0.355124 | -4.05788 | 2.37E-12 | 2.80E-11 |
| SPINK6 | 5.073904 | 0.3181 | -3.99554 | 1.38E-06 | 5.23E-06 |
| FGF20 | 4.046781 | 0.257498 | -3.97414 | 2.75E-16 | 7.51E-15 |
| CRYBA4 | 3.244345 | 0.228551 | -3.82734 | 2.82E-09 | 1.79E-08 |
| SPRR2F | 1.467123 | 0.104094 | -3.81703 | 6.08E-05 | 0.000166 |
| MUCL1 | 13.56492 | 1.051551 | -3.68929 | 7.53E-14 | 1.22E-12 |
| CA6 | 1.130019 | 0.092752 | -3.60683 | 1.29E-09 | 8.78E-09 |
| MYH7B | 6.062776 | 0.512302 | -3.56491 | 0.027206 | 0.041474 |
| VSIG8 | 3.686277 | 0.332283 | -3.47168 | 1.68E-07 | 7.61E-07 |
| FGF19 | 6.451361 | 0.609343 | -3.40428 | 1.52E-14 | 2.83E-13 |
| BPIFB2 | 6.472577 | 0.620437 | -3.38298 | 1.19E-17 | 4.28E-16 |
| PMCH | 1.349533 | 0.140436 | -3.26447 | 0.006788 | 0.011953 |
| SNTN | 3.069016 | 0.323776 | -3.24471 | 7.07E-10 | 5.02E-09 |
| SERPINA6 | 12.55807 | 1.346545 | -3.22128 | 8.36E-14 | 1.34E-12 |
| GP2 | 4.365844 | 0.476493 | -3.19573 | 9.79E-08 | 4.65E-07 |
| C6orf15 | 16.07955 | 1.76323 | -3.18894 | 1.57E-05 | 4.83E-05 |
| PLA2G4D | 0.520597 | 0.057537 | -3.1776 | 0.001141 | 0.002392 |
| SCGB3A1 | 175.0163 | 19.71012 | -3.15048 | 4.70E-12 | 5.21E-11 |
| MROH9 | 0.529063 | 0.060902 | -3.11887 | 1.69E-12 | 2.05E-11 |
| MUC6 | 16.60226 | 1.912137 | -3.11812 | 5.68E-08 | 2.80E-07 |
| FAM216B | 6.800503 | 0.783478 | -3.11768 | 6.71E-13 | 8.95E-12 |
| SCGB2A2 | 56.51972 | 6.640244 | -3.08945 | 1.56E-21 | 1.49E-19 |
| CNGA4 | 3.081578 | 0.366205 | -3.07295 | 2.41E-14 | 4.29E-13 |
| CST2 | 9.084873 | 1.088993 | -3.06047 | 1.94E-11 | 1.90E-10 |
| HPR | 0.649327 | 0.078374 | -3.0505 | 1.54E-06 | 5.75E-06 |
| CCDC60 | 1.822988 | 0.221231 | -3.04268 | 6.56E-13 | 8.77E-12 |
| C6 | 1.653317 | 0.20326 | -3.02397 | 2.19E-05 | 6.51E-05 |
| OVGP1 | 87.85811 | 10.84618 | -3.01799 | 9.40E-11 | 8.04E-10 |
| MS4A8 | 21.4214 | 2.765403 | -2.95349 | 0.000221 | 0.000537 |
| CST1 | 160.1836 | 20.70609 | -2.9516 | 6.73E-10 | 4.81E-09 |
| APOBEC4 | 2.143684 | 0.282011 | -2.92627 | 1.11E-13 | 1.73E-12 |
| S100A3 | 17.27192 | 2.304067 | -2.90617 | 0.000395 | 0.000914 |
| L1TD1 | 0.372798 | 0.049975 | -2.89912 | 0.016379 | 0.026299 |
| RASGRF1 | 2.078801 | 0.279104 | -2.89688 | 2.37E-18 | 1.03E-16 |
| SHISA6 | 1.159173 | 0.156521 | -2.88867 | 1.95E-21 | 1.85E-19 |
| CDHR4 | 10.1779 | 1.385588 | -2.87687 | 7.76E-13 | 1.02E-11 |
| LRRC18 | 1.635598 | 0.223973 | -2.86842 | 4.44E-13 | 6.18E-12 |
| SPRR1B | 17.68375 | 2.450877 | -2.85105 | 1.04E-05 | 3.31E-05 |
| DPEP1 | 8.5088 | 1.179683 | -2.85056 | 4.76E-11 | 4.29E-10 |
| OMG | 5.367901 | 0.744925 | -2.84919 | 3.30E-16 | 8.75E-15 |
| SPRR1A | 13.83482 | 1.926908 | -2.84394 | 0.002077 | 0.004119 |
| PPBP | 1.649908 | 0.238214 | -2.79206 | 0.00355 | 0.006676 |
| STOML3 | 3.732744 | 0.540698 | -2.78734 | 1.94E-09 | 1.27E-08 |
| MUC7 | 0.683321 | 0.099005 | -2.78699 | 2.63E-10 | 2.05E-09 |
| FAM92B | 8.070175 | 1.18461 | -2.76819 | 4.47E-10 | 3.30E-09 |
| TMPRSS11E | 0.641356 | 0.094765 | -2.75869 | 2.47E-06 | 8.89E-06 |
| ABHD12B | 1.341129 | 0.200868 | -2.73913 | 7.12E-11 | 6.22E-10 |
| DMBT1 | 6.680707 | 1.012126 | -2.72261 | 1.39E-14 | 2.60E-13 |
| SRD5A2 | 1.689229 | 0.25604 | -2.72193 | 4.37E-12 | 4.88E-11 |
| GSTA3 | 2.250754 | 0.350412 | -2.68329 | 1.92E-08 | 1.04E-07 |
| KRT36 | 0.502519 | 0.08014 | -2.64859 | 0.012304 | 0.020333 |
| CDX2 | 1.328643 | 0.21363 | -2.63677 | 1.17E-15 | 2.70E-14 |
| GAS2L2 | 4.074611 | 0.656777 | -2.63319 | 1.22E-12 | 1.53E-11 |
| FGF3 | 8.033196 | 1.314498 | -2.61146 | 4.65E-07 | 1.93E-06 |
| DNAI2 | 3.344816 | 0.547482 | -2.61104 | 2.12E-07 | 9.41E-07 |
| CATSPERD | 1.013131 | 0.167521 | -2.59641 | 5.42E-10 | 3.93E-09 |
| ANKRD66 | 1.622669 | 0.269369 | -2.59071 | 2.96E-11 | 2.79E-10 |
| CLCA2 | 0.416391 | 0.069953 | -2.57349 | 1.79E-11 | 1.77E-10 |
| TCTE1 | 1.76243 | 0.297195 | -2.56809 | 3.19E-11 | 2.99E-10 |
| ANKFN1 | 0.681629 | 0.11527 | -2.56397 | 5.62E-31 | 6.96E-28 |
| WDR38 | 17.97368 | 3.055099 | -2.55659 | 8.74E-09 | 5.05E-08 |
| DTHD1 | 0.77224 | 0.131291 | -2.55628 | 2.74E-12 | 3.19E-11 |
| SEZ6L | 1.821332 | 0.314439 | -2.53414 | 6.61E-07 | 2.64E-06 |
| C15orf26 | 3.525122 | 0.611137 | -2.52811 | 9.55E-11 | 8.15E-10 |
| CCDC17 | 6.473018 | 1.135864 | -2.51065 | 9.35E-06 | 3.00E-05 |
| SPRR3 | 9.843566 | 1.742487 | -2.49803 | 1.46E-06 | 5.48E-06 |
| SLC6A2 | 1.114094 | 0.197318 | -2.49727 | 4.24E-13 | 5.91E-12 |
| C1orf189 | 7.724396 | 1.370008 | -2.49524 | 8.88E-10 | 6.17E-09 |
| C9orf24 | 26.06789 | 4.625962 | -2.49445 | 2.72E-09 | 1.73E-08 |
| NKD1 | 6.796495 | 1.213116 | -2.48607 | 7.77E-07 | 3.07E-06 |
| TEKT4 | 3.996414 | 0.716276 | -2.48012 | 4.90E-10 | 3.58E-09 |
| ERICH3 | 2.183068 | 0.392817 | -2.47443 | 6.37E-24 | 1.20E-21 |
| AZU1 | 1.071317 | 0.193521 | -2.46882 | 4.36E-14 | 7.43E-13 |
| CRYBB1 | 5.212671 | 0.951195 | -2.45421 | 1.71E-05 | 5.20E-05 |
| C1orf110 | 0.328765 | 0.060013 | -2.4537 | 1.48E-09 | 9.88E-09 |
| C22orf15 | 2.498834 | 0.456856 | -2.45144 | 2.58E-11 | 2.46E-10 |
| KRT6A | 29.55567 | 5.451704 | -2.43866 | 1.20E-06 | 4.60E-06 |
| DNAH9 | 1.778818 | 0.329557 | -2.43232 | 4.96E-15 | 1.01E-13 |
| MMP26 | 13.37206 | 2.50927 | -2.41388 | 1.87E-15 | 4.13E-14 |
| C1orf87 | 1.115843 | 0.211008 | -2.40277 | 0.036796 | 0.054443 |
| KRT16 | 22.06386 | 4.175771 | -2.40157 | 2.67E-05 | 7.84E-05 |
| ENSG00000226321 | 2.230817 | 0.424379 | -2.39415 | 1.08E-08 | 6.14E-08 |
| CDHR3 | 3.735164 | 0.7161 | -2.38294 | 2.81E-10 | 2.18E-09 |
| FAM166B | 17.05315 | 3.274892 | -2.38052 | 3.46E-11 | 3.22E-10 |
| DNAAF1 | 4.571735 | 0.881294 | -2.37505 | 2.16E-14 | 3.89E-13 |
| ASCL5 | 1.662776 | 0.321331 | -2.37146 | 0.03712 | 0.054861 |
| OBP2B | 24.32147 | 4.705493 | -2.36981 | 8.87E-06 | 2.85E-05 |
| TPPP3 | 88.68487 | 17.18771 | -2.36731 | 1.98E-14 | 3.60E-13 |
| LRRC71 | 4.817065 | 0.93402 | -2.36663 | 5.03E-12 | 5.54E-11 |
| PIH1D3 | 1.155603 | 0.224942 | -2.36102 | 1.19E-10 | 9.96E-10 |
| ADGB | 0.45764 | 0.089376 | -2.35625 | 4.33E-07 | 1.81E-06 |
| LRRC46 | 16.41269 | 3.212553 | -2.35302 | 1.29E-18 | 6.07E-17 |
| SP5 | 16.56241 | 3.244557 | -2.35182 | 6.44E-11 | 5.64E-10 |
| C20orf85 | 83.43747 | 16.37264 | -2.34941 | 7.63E-09 | 4.45E-08 |
| PLG | 0.377379 | 0.074151 | -2.34747 | 0.000218 | 0.000532 |
| ORM1 | 15.97709 | 3.157479 | -2.33916 | 1.14E-07 | 5.36E-07 |
| LRRC15 | 1.072202 | 0.213472 | -2.32846 | 7.37E-06 | 2.41E-05 |
| PZP | 1.567418 | 0.31253 | -2.32632 | 2.50E-14 | 4.41E-13 |
| IHH | 45.47583 | 9.141522 | -2.31459 | 2.25E-27 | 9.89E-25 |
| KCNRG | 2.311393 | 0.467179 | -2.30672 | 6.06E-25 | 1.45E-22 |
| DNAI1 | 4.963516 | 1.016126 | -2.28828 | 1.36E-05 | 4.20E-05 |
| IL5RA | 1.032198 | 0.211361 | -2.28794 | 6.48E-09 | 3.84E-08 |
| TTC29 | 1.356539 | 0.2778 | -2.28781 | 9.22E-10 | 6.39E-09 |
| C4BPA | 18.16067 | 3.740726 | -2.27943 | 1.95E-15 | 4.29E-14 |
| DBH | 1.29951 | 0.268524 | -2.27485 | 1.34E-07 | 6.21E-07 |
| LPPR3 | 3.407065 | 0.713555 | -2.25543 | 7.10E-14 | 1.16E-12 |
| TRIM55 | 1.339172 | 0.281022 | -2.25259 | 3.99E-07 | 1.68E-06 |
| LMNTD1 | 0.32158 | 0.067887 | -2.24397 | 3.79E-14 | 6.51E-13 |
| TEKT1 | 6.664798 | 1.414799 | -2.23596 | 5.65E-06 | 1.89E-05 |
| WFDC6 | 1.326494 | 0.282283 | -2.2324 | 5.31E-14 | 8.90E-13 |
| GPR83 | 0.957345 | 0.20423 | -2.22885 | 2.98E-05 | 8.66E-05 |
| DNAH12 | 0.935761 | 0.201243 | -2.2172 | 2.47E-10 | 1.94E-09 |
| DYNLRB2 | 3.54557 | 0.769386 | -2.20424 | 1.13E-10 | 9.52E-10 |
| FRMPD2 | 0.357227 | 0.078426 | -2.18743 | 8.69E-23 | 1.15E-20 |
| TUBA3E | 1.706638 | 0.379305 | -2.16973 | 3.99E-07 | 1.68E-06 |
| SOX14 | 0.642133 | 0.14334 | -2.16343 | 9.26E-10 | 6.41E-09 |
| FGF8 | 3.040376 | 0.687047 | -2.14577 | 1.00E-06 | 3.89E-06 |
| ADH6 | 1.034131 | 0.233824 | -2.14492 | 2.22E-15 | 4.83E-14 |
| SERPINA3 | 0.647136 | 0.147196 | -2.13633 | 2.42E-14 | 4.31E-13 |
| CFAP74 | 1.010008 | 0.230596 | -2.13093 | 4.55E-10 | 3.35E-09 |
| C1orf194 | 27.22629 | 6.284674 | -2.11509 | 2.35E-12 | 2.78E-11 |
| ENSG00000187905 | 0.592605 | 0.137393 | -2.10876 | 9.62E-09 | 5.52E-08 |
| GDNF | 0.843226 | 0.198468 | -2.08701 | 6.59E-07 | 2.63E-06 |
| MORN5 | 13.22751 | 3.11652 | -2.08553 | 8.67E-07 | 3.40E-06 |
| SLC25A35 | 20.87152 | 4.928731 | -2.08225 | 5.04E-26 | 1.72E-23 |
| PROX1 | 1.188256 | 0.280712 | -2.08168 | 7.38E-13 | 9.75E-12 |
| STMND1 | 2.163194 | 0.511396 | -2.08065 | 5.60E-12 | 6.13E-11 |
| RIIAD1 | 1.97757 | 0.468049 | -2.079 | 7.06E-12 | 7.58E-11 |
| MAP3K19 | 1.288425 | 0.305239 | -2.0776 | 7.24E-10 | 5.13E-09 |
| CHRNA3 | 0.996735 | 0.236328 | -2.07642 | 1.47E-09 | 9.83E-09 |
| ROPN1L | 13.52633 | 3.209387 | -2.0754 | 1.57E-08 | 8.65E-08 |
| LAMP5 | 25.13212 | 5.976121 | -2.07225 | 7.67E-08 | 3.71E-07 |
| CCDC108 | 1.577241 | 0.376388 | -2.06711 | 2.71E-12 | 3.16E-11 |
| FAM154B | 1.981674 | 0.475433 | -2.05941 | 1.46E-13 | 2.22E-12 |
| AKAP14 | 3.649424 | 0.880905 | -2.05061 | 2.42E-08 | 1.28E-07 |
| C9orf171 | 7.268586 | 1.761443 | -2.04492 | 5.56E-09 | 3.35E-08 |
| C1orf64 | 3.146884 | 0.766847 | -2.03691 | 1.47E-20 | 1.09E-18 |
| CFAP99 | 1.006749 | 0.247325 | -2.02523 | 1.29E-09 | 8.74E-09 |
| C11orf88 | 2.504708 | 0.620811 | -2.01242 | 0.000841 | 0.001819 |
| GAD1 | 6.310282 | 1.565489 | -2.01109 | 3.27E-23 | 5.00E-21 |
| XG | 4.66621 | 1.157679 | -2.01102 | 4.80E-20 | 3.27E-18 |
| TMEM212 | 0.464197 | 0.115213 | -2.01043 | 5.16E-06 | 1.74E-05 |
| DNAH10 | 0.584637 | 0.145376 | -2.00775 | 4.57E-13 | 6.35E-12 |
| BMP4 | 11.0823 | 2.77096 | -1.9998 | 3.99E-08 | 2.02E-07 |
| DNAH3 | 0.92899 | 0.233962 | -1.98939 | 5.14E-10 | 3.75E-09 |
| DKK1 | 15.40385 | 3.895522 | -1.9834 | 0.000977 | 0.00208 |
| TMEM190 | 16.49366 | 4.187184 | -1.97786 | 3.31E-05 | 9.55E-05 |
| SCGB2A1 | 1381.112 | 351.1273 | -1.97576 | 7.08E-22 | 7.65E-20 |
| KRT6B | 4.047143 | 1.029762 | -1.97459 | 2.98E-05 | 8.67E-05 |
| SERPINA11 | 4.167559 | 1.060423 | -1.97456 | 7.67E-18 | 2.85E-16 |
| ENSG00000273259 | 0.346933 | 0.088933 | -1.96386 | 1.79E-13 | 2.67E-12 |
| ENKUR | 4.408553 | 1.131958 | -1.96148 | 1.34E-08 | 7.48E-08 |
| VWA3B | 1.416064 | 0.364352 | -1.95848 | 2.46E-10 | 1.93E-09 |
| CCDC78 | 11.13673 | 2.866659 | -1.95788 | 1.40E-06 | 5.29E-06 |
| TTLL10 | 2.171644 | 0.561507 | -1.95141 | 6.38E-07 | 2.56E-06 |
| ENSG00000188396 | 2.96378 | 0.766585 | -1.95092 | 4.74E-08 | 2.38E-07 |
| TEKT3 | 1.050527 | 0.271814 | -1.95042 | 8.61E-11 | 7.40E-10 |
| GREM2 | 5.735789 | 1.48543 | -1.94911 | 6.31E-12 | 6.85E-11 |
| ODAM | 8.160081 | 2.121778 | -1.94331 | 0.003185 | 0.006055 |
| CCDC42B | 8.088352 | 2.133994 | -1.92229 | 3.99E-08 | 2.02E-07 |
| C1orf158 | 2.331432 | 0.616442 | -1.91918 | 4.32E-07 | 1.80E-06 |
| PIP | 3.870216 | 1.028951 | -1.91124 | 1.85E-11 | 1.82E-10 |
| LRRC36 | 0.961673 | 0.257774 | -1.89944 | 4.78E-15 | 9.77E-14 |
| ORM2 | 8.183088 | 2.195628 | -1.89801 | 5.62E-13 | 7.64E-12 |
| DCDC2B | 0.757244 | 0.20368 | -1.89445 | 1.50E-05 | 4.62E-05 |
| SPATA18 | 9.043699 | 2.434611 | -1.89322 | 3.95E-29 | 3.36E-26 |
| EPPIN | 0.354298 | 0.095474 | -1.89179 | 3.87E-13 | 5.43E-12 |
| MUC5AC | 3.268906 | 0.883625 | -1.8873 | 0.020725 | 0.032517 |
| C4orf22 | 0.324823 | 0.087899 | -1.88574 | 1.88E-07 | 8.43E-07 |
| TUBA3D | 4.09982 | 1.111529 | -1.88301 | 2.00E-05 | 6.00E-05 |
| C2orf73 | 0.496887 | 0.135151 | -1.87834 | 8.82E-05 | 0.000233 |
| ATP4B | 0.441966 | 0.120476 | -1.87519 | 0.005039 | 0.009131 |
| C11orf16 | 2.511656 | 0.684857 | -1.87476 | 4.41E-10 | 3.27E-09 |
| SPINK5 | 3.991343 | 1.09156 | -1.87048 | 8.03E-05 | 0.000214 |
| CFAP52 | 2.262989 | 0.623467 | -1.85984 | 0.002612 | 0.00506 |
| SMIM6 | 17.29132 | 4.787069 | -1.85283 | 5.33E-15 | 1.08E-13 |
| KIF19 | 2.373861 | 0.66014 | -1.84639 | 5.91E-06 | 1.97E-05 |
| PTCHD4 | 0.353231 | 0.098329 | -1.84492 | 1.16E-16 | 3.40E-15 |
| SLC47A1 | 20.50862 | 5.712657 | -1.844 | 2.72E-21 | 2.51E-19 |
| SCGB1D1 | 31.63229 | 8.828108 | -1.84122 | 0.015855 | 0.025515 |
| LRRC26 | 6.746886 | 1.883806 | -1.84057 | 2.68E-19 | 1.56E-17 |
| TESC | 21.12013 | 5.934077 | -1.83152 | 5.17E-21 | 4.32E-19 |
| KRTAP3-1 | 1.113213 | 0.313243 | -1.82937 | 0.035143 | 0.052305 |
| FAM179A | 0.548018 | 0.154415 | -1.82741 | 1.72E-06 | 6.38E-06 |
| RSPH4A | 6.447732 | 1.820154 | -1.82473 | 1.64E-08 | 8.99E-08 |
| MUC5B | 31.50114 | 8.89862 | -1.82375 | 3.26E-13 | 4.64E-12 |
| VWA3A | 4.550821 | 1.286888 | -1.82224 | 4.01E-11 | 3.68E-10 |
| CFAP58 | 0.755888 | 0.214162 | -1.81947 | 2.53E-08 | 1.34E-07 |
| ENSG00000234409 | 2.365408 | 0.671516 | -1.8166 | 3.49E-07 | 1.49E-06 |
| CFAP43 | 3.714836 | 1.057111 | -1.81317 | 3.59E-11 | 3.33E-10 |
| PTGS2 | 5.878471 | 1.676041 | -1.81038 | 7.00E-13 | 9.28E-12 |
| FAM183A | 26.72297 | 7.682818 | -1.79837 | 4.10E-08 | 2.08E-07 |
| CXCL14 | 175.1483 | 50.44222 | -1.79587 | 2.48E-18 | 1.06E-16 |
| PPP1R32 | 5.9638 | 1.718933 | -1.79472 | 4.17E-10 | 3.11E-09 |
| ADAMTSL2 | 5.855563 | 1.687928 | -1.79455 | 0.000119 | 0.000306 |
| LCN15 | 1.447334 | 0.418547 | -1.78994 | 1.38E-11 | 1.40E-10 |
| DEFB4A | 2.028139 | 0.590278 | -1.78069 | 1.95E-13 | 2.88E-12 |
| CREB3L1 | 34.9018 | 10.16222 | -1.78009 | 3.85E-22 | 4.45E-20 |
| TSPAN11 | 3.241245 | 0.944351 | -1.77915 | 1.03E-24 | 2.30E-22 |
| FAM159B | 0.778658 | 0.227237 | -1.77679 | 0.038034 | 0.056111 |
| SLC23A1 | 3.057821 | 0.893764 | -1.77454 | 3.39E-09 | 2.12E-08 |
| DLEC1 | 2.049402 | 0.600398 | -1.77121 | 5.77E-08 | 2.84E-07 |
| HP | 20.86129 | 6.160166 | -1.75979 | 2.44E-12 | 2.87E-11 |
| ZNF474 | 1.752819 | 0.518004 | -1.75864 | 1.76E-15 | 3.89E-14 |
| GRIN3B | 1.390769 | 0.411302 | -1.75761 | 9.96E-09 | 5.69E-08 |
| GALNT16 | 3.68346 | 1.090808 | -1.75566 | 4.09E-13 | 5.72E-12 |
| DYDC1 | 0.83701 | 0.2479 | -1.75549 | 0.002146 | 0.004241 |
| LRRC10B | 8.097172 | 2.406895 | -1.75024 | 1.12E-08 | 6.32E-08 |
| LRRC48 | 3.723732 | 1.11552 | -1.73903 | 6.23E-19 | 3.21E-17 |
| TCHH | 0.841493 | 0.254595 | -1.72475 | 0.00244 | 0.004753 |
| SCGB1D2 | 317.0296 | 95.91956 | -1.72472 | 4.10E-10 | 3.06E-09 |
| KRT6C | 0.660753 | 0.200349 | -1.72159 | 0.000627 | 0.001392 |
| APCDD1 | 53.65554 | 16.36385 | -1.71321 | 2.34E-07 | 1.03E-06 |
| NEK10 | 0.508593 | 0.15523 | -1.71211 | 1.99E-11 | 1.94E-10 |
| IL17C | 0.766091 | 0.23539 | -1.70247 | 0.001637 | 0.00332 |
| LDLRAD1 | 7.960781 | 2.455565 | -1.69685 | 1.40E-06 | 5.28E-06 |
| GLYATL3 | 1.292467 | 0.399366 | -1.69435 | 2.43E-06 | 8.77E-06 |
| CCDC170 | 5.955304 | 1.843335 | -1.69186 | 1.91E-18 | 8.45E-17 |
| FGL1 | 0.690654 | 0.214978 | -1.68378 | 1.12E-17 | 4.05E-16 |
| CAPSL | 19.23714 | 6.010191 | -1.67841 | 2.99E-07 | 1.29E-06 |
| CEL | 7.526727 | 2.352025 | -1.67812 | 1.52E-11 | 1.53E-10 |
| C6orf118 | 3.60208 | 1.132839 | -1.66889 | 1.30E-06 | 4.94E-06 |
| RSPH1 | 20.56215 | 6.477057 | -1.66658 | 4.36E-12 | 4.86E-11 |
| TTC25 | 6.75359 | 2.139775 | -1.6582 | 5.42E-11 | 4.82E-10 |
| WIF1 | 23.45465 | 7.43509 | -1.65745 | 1.41E-18 | 6.56E-17 |
| LMX1A | 0.280099 | 0.088929 | -1.65522 | 5.36E-10 | 3.89E-09 |
| IQUB | 0.73223 | 0.23684 | -1.62838 | 3.62E-10 | 2.74E-09 |
| ART3 | 0.404692 | 0.130983 | -1.62745 | 1.13E-15 | 2.61E-14 |
| C10orf107 | 4.88478 | 1.582429 | -1.62615 | 2.62E-12 | 3.06E-11 |
| FMO2 | 1.333799 | 0.435129 | -1.61603 | 0.001231 | 0.00256 |
| CFAP61 | 0.667401 | 0.218195 | -1.61293 | 2.30E-05 | 6.82E-05 |
| COL17A1 | 2.655296 | 0.868972 | -1.61149 | 7.90E-19 | 3.91E-17 |
| ITLN2 | 1.13066 | 0.370621 | -1.60915 | 1.03E-09 | 7.07E-09 |
| KRT13 | 5.524477 | 1.812546 | -1.60782 | 0.026357 | 0.040307 |
| PGR | 15.15173 | 4.981362 | -1.60487 | 4.95E-20 | 3.32E-18 |
| ZMYND10 | 17.07006 | 5.615757 | -1.60392 | 6.84E-09 | 4.02E-08 |
| IL19 | 2.542878 | 0.839145 | -1.59947 | 3.18E-09 | 2.00E-08 |
| TMEM232 | 0.55273 | 0.182974 | -1.59494 | 3.72E-08 | 1.90E-07 |
| CATIP | 1.937675 | 0.650518 | -1.57467 | 1.85E-05 | 5.57E-05 |
| ACSM1 | 0.947718 | 0.319219 | -1.56991 | 1.88E-11 | 1.84E-10 |
| EFCAB1 | 4.010217 | 1.351326 | -1.5693 | 2.79E-05 | 8.15E-05 |
| DRC7 | 2.970398 | 1.002803 | -1.56662 | 7.39E-07 | 2.93E-06 |
| TMEM210 | 0.444541 | 0.150405 | -1.56347 | 1.57E-16 | 4.48E-15 |
| CCL22 | 2.474192 | 0.837973 | -1.56198 | 1.32E-11 | 1.34E-10 |
| ARMC4 | 1.33185 | 0.451621 | -1.56025 | 6.66E-07 | 2.66E-06 |
| C5orf49 | 12.53964 | 4.257087 | -1.55856 | 0.000478 | 0.001087 |
| FGFBP1 | 9.144812 | 3.104942 | -1.55839 | 8.33E-06 | 2.70E-05 |
| TMEM114 | 0.497102 | 0.169196 | -1.55484 | 3.99E-15 | 8.29E-14 |
| PIGR | 402.4563 | 137.0389 | -1.55425 | 3.03E-17 | 9.66E-16 |
| MYCBPAP | 0.709567 | 0.241743 | -1.55346 | 5.02E-13 | 6.90E-12 |
| CSF3 | 3.648496 | 1.244645 | -1.55157 | 4.20E-16 | 1.09E-14 |
| KMO | 0.852535 | 0.291418 | -1.54867 | 1.61E-07 | 7.30E-07 |
| RRAD | 18.31951 | 6.265575 | -1.54786 | 0.000145 | 0.000367 |
| LTF | 229.8678 | 78.64974 | -1.54729 | 0.000325 | 0.000766 |
| TFF3 | 722.965 | 248.0372 | -1.54337 | 8.54E-25 | 1.97E-22 |
| HES5 | 0.807667 | 0.27866 | -1.53526 | 3.63E-23 | 5.50E-21 |
| CH507-396I9.6 | 0.308699 | 0.106513 | -1.53517 | 6.54E-07 | 2.62E-06 |
| NHLRC4 | 4.082426 | 1.422509 | -1.52099 | 1.59E-11 | 1.59E-10 |
| SERPINA5 | 43.52688 | 15.29036 | -1.50928 | 3.63E-12 | 4.12E-11 |
| SPDEF | 103.5011 | 36.47051 | -1.50484 | 5.85E-27 | 2.34E-24 |
| ENPP3 | 8.864072 | 3.126878 | -1.50325 | 5.90E-17 | 1.80E-15 |
| C9orf117 | 5.352886 | 1.890902 | -1.50124 | 1.71E-11 | 1.69E-10 |
| XXbac-BPG32J3.19 | 0.289613 | 0.102549 | -1.49781 | 0.020028 | 0.031545 |
| WDR63 | 1.759647 | 0.624106 | -1.49542 | 1.74E-05 | 5.27E-05 |
| ZCCHC12 | 4.534562 | 1.608792 | -1.49499 | 1.47E-05 | 4.54E-05 |
| SCGB1A1 | 26.73216 | 9.504247 | -1.49193 | 2.87E-05 | 8.38E-05 |
| NPTX1 | 0.447939 | 0.159396 | -1.49069 | 2.56E-07 | 1.12E-06 |
| CAPN6 | 32.3529 | 11.51316 | -1.49061 | 1.41E-10 | 1.16E-09 |
| NT5E | 21.16784 | 7.541525 | -1.48895 | 3.88E-17 | 1.22E-15 |
| CYP24A1 | 3.253137 | 1.159263 | -1.48862 | 1.28E-07 | 5.96E-07 |
| SERPINB3 | 0.981938 | 0.350247 | -1.48726 | 0.001159 | 0.002425 |
| AK8 | 3.817162 | 1.364118 | -1.48453 | 2.14E-16 | 5.99E-15 |
| POMC | 3.930449 | 1.404741 | -1.48439 | 0.000147 | 0.000371 |
| TFF1 | 10.43966 | 3.746999 | -1.47827 | 9.26E-16 | 2.22E-14 |
| FAM189A2 | 6.521372 | 2.340952 | -1.47808 | 2.92E-16 | 7.91E-15 |
| SERPINA4 | 3.241051 | 1.166072 | -1.4748 | 1.74E-16 | 4.91E-15 |
| CFAP57 | 2.650982 | 0.956949 | -1.47001 | 8.78E-09 | 5.07E-08 |
| FABP4 | 11.32718 | 4.095735 | -1.4676 | 6.46E-10 | 4.62E-09 |
| PRR29 | 4.357706 | 1.579107 | -1.46446 | 0.000265 | 0.000635 |
| KIAA1324 | 73.82315 | 26.91464 | -1.45568 | 1.12E-25 | 3.33E-23 |
| IL20RA | 7.013215 | 2.566887 | -1.45006 | 5.57E-28 | 3.14E-25 |
| ACSL5 | 38.99839 | 14.29499 | -1.4479 | 3.88E-14 | 6.66E-13 |
| PIFO | 12.65521 | 4.646496 | -1.44552 | 2.38E-09 | 1.53E-08 |
| SLC43A1 | 17.75181 | 6.537341 | -1.44119 | 1.13E-18 | 5.41E-17 |
| FAM163B | 0.478584 | 0.176532 | -1.43885 | 0.004465 | 0.008185 |
| SLC46A2 | 0.937119 | 0.347563 | -1.43096 | 1.49E-16 | 4.28E-15 |
| HDC | 0.552723 | 0.205571 | -1.42692 | 0.00132 | 0.002732 |
| ENO4 | 0.878588 | 0.326789 | -1.42683 | 2.26E-08 | 1.21E-07 |
| AXIN2 | 9.974528 | 3.714984 | -1.42489 | 1.19E-11 | 1.23E-10 |
| ANKK1 | 0.761827 | 0.284305 | -1.42202 | 3.12E-11 | 2.94E-10 |
| KRT24 | 0.425446 | 0.159152 | -1.41857 | 7.72E-05 | 0.000206 |
| KRT1 | 0.367759 | 0.137737 | -1.41684 | 0.001266 | 0.002625 |
| C7orf57 | 2.104037 | 0.788232 | -1.41647 | 6.25E-05 | 0.000171 |
| ECT2L | 0.325292 | 0.122417 | -1.40993 | 3.94E-05 | 0.000112 |
| PIK3C2G | 0.48337 | 0.181954 | -1.40955 | 8.68E-06 | 2.80E-05 |
| BAMBI | 28.39753 | 10.72776 | -1.40442 | 1.78E-12 | 2.16E-11 |
| MSX1 | 325.4874 | 123.1106 | -1.40265 | 3.41E-21 | 3.05E-19 |
| CCDC65 | 6.341804 | 2.401625 | -1.40088 | 0.000184 | 0.000456 |
| SPTSSB | 2.191956 | 0.832306 | -1.39703 | 3.08E-13 | 4.40E-12 |
| CFAP53 | 6.333106 | 2.408444 | -1.39481 | 3.58E-08 | 1.84E-07 |
| FOXL1 | 0.577662 | 0.220575 | -1.38896 | 3.08E-15 | 6.54E-14 |
| CDC20B | 6.258729 | 2.39384 | -1.38654 | 4.29E-09 | 2.65E-08 |
| EDN3 | 14.54407 | 5.564441 | -1.38612 | 3.04E-14 | 5.30E-13 |
| EDAR | 1.355336 | 0.520365 | -1.38106 | 1.42E-07 | 6.51E-07 |
| C16orf71 | 1.64394 | 0.631535 | -1.38022 | 1.31E-15 | 2.99E-14 |
| ADAMTS8 | 7.744411 | 2.977027 | -1.37928 | 3.45E-12 | 3.94E-11 |
| LRRC23 | 15.24732 | 5.862254 | -1.37903 | 1.26E-09 | 8.55E-09 |
| AGBL2 | 1.198156 | 0.460876 | -1.37836 | 1.06E-05 | 3.37E-05 |
| ADAMTS19 | 2.29309 | 0.88376 | -1.37557 | 5.14E-16 | 1.31E-14 |
| ANKDD1B | 0.853982 | 0.329713 | -1.373 | 3.24E-08 | 1.68E-07 |
| SPAG8 | 3.979528 | 1.537579 | -1.37194 | 7.35E-07 | 2.91E-06 |
| DNAH6 | 1.114797 | 0.430974 | -1.37111 | 3.74E-06 | 1.30E-05 |
| TSNAXIP1 | 3.595144 | 1.39063 | -1.37031 | 5.89E-12 | 6.43E-11 |
| CD164L2 | 3.984965 | 1.542692 | -1.36912 | 5.67E-05 | 0.000156 |
| CFAP46 | 0.970361 | 0.375975 | -1.36788 | 3.01E-06 | 1.06E-05 |
| DRC1 | 5.787598 | 2.242475 | -1.36787 | 0.000223 | 0.000543 |
| EFCAB10 | 1.591328 | 0.617128 | -1.36659 | 6.30E-09 | 3.74E-08 |
| OGN | 1.190205 | 0.461825 | -1.36579 | 0.000385 | 0.000893 |
| C9orf116 | 17.62961 | 6.904018 | -1.35249 | 7.28E-19 | 3.69E-17 |
| C5AR2 | 1.001552 | 0.393219 | -1.34883 | 4.10E-22 | 4.65E-20 |
| SLC6A14 | 2.829208 | 1.111056 | -1.34847 | 3.77E-19 | 2.09E-17 |
| PACRG | 3.71479 | 1.470989 | -1.33649 | 5.55E-08 | 2.75E-07 |
| SERPINA1 | 124.6706 | 49.54244 | -1.33138 | 1.26E-12 | 1.57E-11 |
| ZDHHC1 | 12.12197 | 4.818096 | -1.33109 | 2.66E-34 | 1.81E-30 |
| UNC93A | 0.940031 | 0.373636 | -1.33107 | 4.70E-07 | 1.95E-06 |
| C9orf152 | 7.641296 | 3.037544 | -1.33091 | 1.09E-25 | 3.31E-23 |
| SMPD3 | 3.078568 | 1.225434 | -1.32897 | 1.14E-27 | 5.54E-25 |
| ARMC3 | 5.317367 | 2.118655 | -1.32756 | 1.16E-09 | 7.93E-09 |
| CFAP45 | 14.11075 | 5.627526 | -1.32622 | 1.70E-09 | 1.13E-08 |
| MB | 3.583164 | 1.430438 | -1.32478 | 1.18E-08 | 6.64E-08 |
| C11orf53 | 0.345092 | 0.137778 | -1.32464 | 0.00193 | 0.003855 |
| CCDC96 | 6.876449 | 2.74859 | -1.32297 | 3.72E-14 | 6.40E-13 |
| HSD17B2 | 0.524363 | 0.209744 | -1.32194 | 3.29E-12 | 3.77E-11 |
| PLD5 | 0.266636 | 0.106962 | -1.31777 | 4.06E-11 | 3.72E-10 |
| RSPH14 | 1.661571 | 0.66776 | -1.31515 | 1.08E-08 | 6.14E-08 |
| TDGF1 | 0.895478 | 0.360083 | -1.31433 | 1.75E-10 | 1.42E-09 |
| SFN | 227.8224 | 91.82247 | -1.31099 | 3.06E-18 | 1.27E-16 |
| UPK1B | 36.34508 | 14.64895 | -1.31096 | 0.00011 | 0.000286 |
| BAIAP3 | 8.228992 | 3.319572 | -1.30972 | 3.40E-07 | 1.45E-06 |
| GIPR | 0.788624 | 0.318226 | -1.30929 | 0.000662 | 0.001459 |
| PLA2G4A | 17.67032 | 7.143835 | -1.30656 | 3.45E-16 | 9.13E-15 |
| C4BPB | 1.03304 | 0.419605 | -1.29979 | 2.38E-14 | 4.24E-13 |
| TMEM26 | 1.349484 | 0.548433 | -1.29902 | 0.000215 | 0.000524 |
| BEX5 | 59.72911 | 24.29716 | -1.29765 | 1.32E-07 | 6.11E-07 |
| TMEM211 | 4.874483 | 1.982943 | -1.29761 | 1.41E-10 | 1.16E-09 |
| CAPS | 224.5074 | 91.38042 | -1.29681 | 1.39E-16 | 4.03E-15 |
| ZNF385B | 2.303375 | 0.938007 | -1.29608 | 3.06E-09 | 1.94E-08 |
| CCBE1 | 0.415103 | 0.169088 | -1.29569 | 0.024015 | 0.037112 |
| CDKN1A | 72.82244 | 29.70592 | -1.29363 | 7.51E-38 | 1.02E-33 |
| FAM81B | 4.142818 | 1.696626 | -1.28794 | 9.02E-07 | 3.52E-06 |
| SLC5A1 | 4.354031 | 1.792326 | -1.28052 | 4.91E-21 | 4.16E-19 |
| TTC16 | 0.404219 | 0.166604 | -1.27871 | 5.50E-08 | 2.73E-07 |
| TNS4 | 2.759644 | 1.137819 | -1.27821 | 2.13E-13 | 3.13E-12 |
| RIBC1 | 5.578395 | 2.300365 | -1.27799 | 2.84E-14 | 4.97E-13 |
| PLEKHG7 | 0.822474 | 0.33924 | -1.27766 | 6.90E-13 | 9.16E-12 |
| NME9 | 1.210171 | 0.500288 | -1.27438 | 2.06E-05 | 6.15E-05 |
| NXF3 | 0.810856 | 0.335301 | -1.27399 | 4.86E-10 | 3.56E-09 |
| CCDC113 | 6.11148 | 2.527419 | -1.27386 | 1.87E-13 | 2.76E-12 |
| CCDC37 | 2.647466 | 1.095755 | -1.27269 | 0.000627 | 0.001391 |
| TREH | 0.461718 | 0.191466 | -1.26993 | 1.11E-11 | 1.16E-10 |
| PHLDA1 | 15.72354 | 6.526824 | -1.26847 | 2.97E-17 | 9.51E-16 |
| TSPAN8 | 10.15161 | 4.217508 | -1.26725 | 1.89E-13 | 2.79E-12 |
| CCDC129 | 0.569983 | 0.236982 | -1.26614 | 4.36E-13 | 6.07E-12 |
| AK7 | 3.055706 | 1.273301 | -1.26293 | 6.71E-08 | 3.28E-07 |
| C11orf70 | 4.246793 | 1.773057 | -1.26014 | 4.52E-05 | 0.000127 |
| CCL24 | 1.504181 | 0.628671 | -1.2586 | 2.81E-11 | 2.66E-10 |
| PLEKHS1 | 12.45016 | 5.210443 | -1.25669 | 1.25E-17 | 4.44E-16 |
| C9orf9 | 7.602894 | 3.183242 | -1.25605 | 7.14E-23 | 9.82E-21 |
| ONECUT3 | 0.267999 | 0.112376 | -1.2539 | 9.17E-20 | 5.86E-18 |
| ZBBX | 1.995117 | 0.840381 | -1.24736 | 0.002093 | 0.004148 |
| FOXA2 | 22.14398 | 9.329128 | -1.2471 | 3.72E-23 | 5.57E-21 |
| CXCL3 | 4.75762 | 2.019428 | -1.23629 | 1.07E-12 | 1.37E-11 |
| ENSG00000260220 | 1.149749 | 0.488399 | -1.23519 | 0.023364 | 0.036209 |
| C3orf36 | 0.865402 | 0.368303 | -1.23248 | 2.93E-13 | 4.19E-12 |
| SPEF1 | 8.238449 | 3.522993 | -1.22557 | 7.89E-05 | 0.000211 |
| CLCA4 | 0.380779 | 0.16376 | -1.21737 | 3.61E-10 | 2.74E-09 |
| TMED6 | 2.026944 | 0.872383 | -1.21627 | 1.29E-18 | 6.07E-17 |
| CFAP221 | 1.577766 | 0.683334 | -1.20722 | 1.09E-05 | 3.45E-05 |
| PPP1R42 | 0.849134 | 0.368073 | -1.206 | 0.000165 | 0.000412 |
| VWA2 | 6.195966 | 2.685781 | -1.20599 | 5.67E-05 | 0.000156 |
| PF4V1 | 1.453086 | 0.630947 | -1.20353 | 0.023659 | 0.036616 |
| GSDMA | 0.490606 | 0.213116 | -1.20293 | 0.008589 | 0.01478 |
| LRRC43 | 2.744162 | 1.197067 | -1.19686 | 1.70E-07 | 7.67E-07 |
| WDR78 | 2.77351 | 1.210278 | -1.19637 | 8.81E-10 | 6.13E-09 |
| DNAH11 | 0.881043 | 0.384993 | -1.19438 | 1.50E-13 | 2.27E-12 |
| NME5 | 5.655163 | 2.475331 | -1.19195 | 6.12E-17 | 1.86E-15 |
| FZD10 | 17.04216 | 7.461573 | -1.19156 | 5.38E-16 | 1.36E-14 |
| LINC00116 | 30.48026 | 13.34943 | -1.1911 | 0.012228 | 0.02022 |
| FUT6 | 1.887292 | 0.827756 | -1.18904 | 3.06E-09 | 1.94E-08 |
| SEC14L5 | 0.429255 | 0.188608 | -1.18644 | 6.78E-06 | 2.23E-05 |
| TNFSF14 | 1.375293 | 0.604318 | -1.18636 | 1.01E-10 | 8.57E-10 |
| CCDC33 | 1.906986 | 0.838758 | -1.18497 | 8.51E-05 | 0.000225 |
| WDR66 | 1.983264 | 0.876036 | -1.17881 | 2.38E-12 | 2.81E-11 |
| GRM8 | 0.442179 | 0.195451 | -1.17783 | 0.000886 | 0.001906 |
| DZIP1L | 1.586334 | 0.701817 | -1.17653 | 0.001217 | 0.002534 |
| SYCE1L | 7.023031 | 3.114681 | -1.17301 | 1.27E-13 | 1.97E-12 |
| TP73 | 2.90618 | 1.29108 | -1.17055 | 7.64E-20 | 4.91E-18 |
| FHAD1 | 1.15381 | 0.51478 | -1.16438 | 0.000424 | 0.000976 |
| DUOXA1 | 1.742115 | 0.779304 | -1.16058 | 6.07E-05 | 0.000166 |
| TNFSF8 | 0.73223 | 0.3284 | -1.15684 | 0.006176 | 0.010983 |
| WDR49 | 1.02763 | 0.462336 | -1.15231 | 5.05E-05 | 0.000141 |
| HPX | 0.892654 | 0.403528 | -1.14543 | 5.38E-10 | 3.91E-09 |
| GJB2 | 18.72052 | 8.47838 | -1.14276 | 2.09E-09 | 1.36E-08 |
| ECM1 | 18.6927 | 8.469916 | -1.14206 | 1.03E-10 | 8.73E-10 |
| SHH | 2.50007 | 1.133033 | -1.14178 | 1.66E-13 | 2.49E-12 |
| APOD | 16.37421 | 7.431417 | -1.13972 | 0.000138 | 0.000351 |
| ANAPC4 | 11.53804 | 5.245821 | -1.13716 | 4.79E-14 | 8.10E-13 |
| DIO2 | 7.43997 | 3.386999 | -1.13529 | 5.77E-08 | 2.84E-07 |
| SAA2 | 8.425801 | 3.836531 | -1.13501 | 0.00012 | 0.000309 |
| KCNK6 | 9.024363 | 4.11201 | -1.13398 | 5.67E-21 | 4.68E-19 |
| CEMIP | 4.704016 | 2.143921 | -1.13364 | 2.64E-08 | 1.39E-07 |
| OMD | 0.257389 | 0.117321 | -1.13349 | 6.60E-06 | 2.18E-05 |
| TMEM150C | 4.584234 | 2.09494 | -1.12977 | 1.77E-13 | 2.63E-12 |
| TMEM233 | 0.853548 | 0.390204 | -1.12924 | 0.000656 | 0.001448 |
| AGR3 | 26.32165 | 12.03794 | -1.12866 | 1.21E-06 | 4.63E-06 |
| SORBS2 | 4.328425 | 1.979619 | -1.12862 | 9.95E-29 | 6.78E-26 |
| CES3 | 6.237445 | 2.858026 | -1.12594 | 9.61E-10 | 6.63E-09 |
| GRIN1 | 0.542848 | 0.249052 | -1.1241 | 7.34E-17 | 2.21E-15 |
| EN2 | 3.020176 | 1.388167 | -1.12145 | 3.74E-08 | 1.91E-07 |
| KLKB1 | 0.490011 | 0.225259 | -1.12123 | 3.06E-07 | 1.31E-06 |
| NWD1 | 1.992243 | 0.915914 | -1.12111 | 2.06E-14 | 3.73E-13 |
| ENTPD3 | 9.084314 | 4.180519 | -1.1197 | 1.05E-20 | 8.01E-19 |
| FAM47E | 0.503662 | 0.23196 | -1.11858 | 1.61E-11 | 1.61E-10 |
| TMC5 | 9.464899 | 4.361618 | -1.11772 | 1.30E-18 | 6.11E-17 |
| FMO5 | 1.359927 | 0.626804 | -1.11744 | 1.63E-11 | 1.63E-10 |
| PKDCC | 26.57422 | 12.2775 | -1.11401 | 6.19E-13 | 8.30E-12 |
| CCDC13 | 0.814338 | 0.37632 | -1.11367 | 7.49E-05 | 0.000201 |
| COL2A1 | 7.962028 | 3.682127 | -1.1126 | 0.019852 | 0.031279 |
| CXCL5 | 9.766904 | 4.517237 | -1.11246 | 1.95E-08 | 1.05E-07 |
| FANK1 | 5.448504 | 2.520294 | -1.11227 | 1.44E-12 | 1.78E-11 |
| EPHA7 | 3.852963 | 1.783638 | -1.11115 | 3.56E-06 | 1.24E-05 |
| CXCL2 | 8.144409 | 3.777041 | -1.10855 | 5.77E-10 | 4.16E-09 |
| EPHA3 | 0.583097 | 0.27072 | -1.10693 | 4.86E-09 | 2.96E-08 |
| CORIN | 0.283357 | 0.131711 | -1.10525 | 1.42E-06 | 5.35E-06 |
| DNAH5 | 0.921167 | 0.42867 | -1.1036 | 0.002664 | 0.005149 |
| BEST4 | 2.369003 | 1.103148 | -1.10265 | 7.98E-06 | 2.59E-05 |
| SLC22A4 | 1.720784 | 0.805609 | -1.09491 | 1.20E-09 | 8.16E-09 |
| CCL20 | 15.669 | 7.341412 | -1.09378 | 9.93E-08 | 4.70E-07 |
| ODF3B | 19.3285 | 9.059886 | -1.09316 | 2.70E-08 | 1.42E-07 |
| FAM150B | 6.17384 | 2.897129 | -1.09154 | 5.01E-09 | 3.04E-08 |
| PCYT1B | 1.475667 | 0.693608 | -1.08917 | 5.18E-08 | 2.58E-07 |
| VMAC | 7.322174 | 3.441689 | -1.08916 | 1.92E-28 | 1.25E-25 |
| C15orf65 | 6.260589 | 2.943826 | -1.08861 | 2.51E-22 | 3.02E-20 |
| ALDH3A1 | 5.615579 | 2.641146 | -1.08827 | 3.36E-06 | 1.17E-05 |
| PPIL6 | 3.84864 | 1.81267 | -1.08623 | 2.65E-05 | 7.78E-05 |
| PPAP2C | 35.63504 | 16.79537 | -1.08523 | 2.48E-31 | 4.23E-28 |
| GCNT1 | 13.1897 | 6.221986 | -1.08396 | 3.91E-19 | 2.15E-17 |
| TMEM89 | 0.388377 | 0.183226 | -1.08383 | 9.15E-05 | 0.000241 |
| RAET1E | 0.273843 | 0.129234 | -1.08337 | 0.005015 | 0.009092 |
| EPHB3 | 29.40218 | 13.87929 | -1.08299 | 0.000537 | 0.001208 |
| CDHR1 | 2.748745 | 1.297931 | -1.08256 | 0.001411 | 0.002899 |
| ADAMTS6 | 1.743051 | 0.823632 | -1.08154 | 5.45E-05 | 0.000151 |
| UBXN10 | 10.3056 | 4.871053 | -1.08112 | 5.98E-15 | 1.20E-13 |
| FBXO15 | 0.570802 | 0.269817 | -1.08101 | 1.40E-07 | 6.41E-07 |
| CPM | 19.27656 | 9.11624 | -1.08034 | 2.92E-06 | 1.03E-05 |
| IGFBP4 | 228.4182 | 108.1826 | -1.07821 | 2.27E-14 | 4.07E-13 |
| DCHS2 | 0.500219 | 0.236943 | -1.07802 | 0.001517 | 0.003098 |
| KCNN4 | 20.57136 | 9.753225 | -1.07669 | 1.70E-11 | 1.68E-10 |
| KRT5 | 42.61197 | 20.22323 | -1.07525 | 0.010592 | 0.017813 |
| WDR93 | 1.278852 | 0.607848 | -1.07307 | 4.03E-07 | 1.69E-06 |
| RASSF6 | 3.35057 | 1.592919 | -1.07273 | 4.02E-21 | 3.47E-19 |
| KLK14 | 7.86147 | 3.738868 | -1.0722 | 0.014978 | 0.024229 |
| FAM149A | 5.746141 | 2.732843 | -1.07219 | 6.96E-18 | 2.63E-16 |
| KIAA0226L | 1.566309 | 0.745055 | -1.07195 | 5.33E-15 | 1.08E-13 |
| MIA | 1.482746 | 0.705387 | -1.07178 | 0.000293 | 0.000696 |
| HHIP | 0.634273 | 0.301795 | -1.07153 | 2.18E-13 | 3.20E-12 |
| TGFBI | 42.86517 | 20.41682 | -1.07005 | 1.68E-06 | 6.27E-06 |
| IQCH | 0.697868 | 0.332463 | -1.06976 | 6.79E-14 | 1.11E-12 |
| S100P | 17.05321 | 8.130777 | -1.06858 | 0.000447 | 0.001024 |
| TPH1 | 3.113578 | 1.484835 | -1.06827 | 0.003634 | 0.006814 |
| CREB3L4 | 35.92071 | 17.14366 | -1.06714 | 5.54E-20 | 3.64E-18 |
| FAM3D | 3.810292 | 1.819068 | -1.0667 | 3.82E-06 | 1.33E-05 |
| TMEM231 | 7.204033 | 3.449505 | -1.06242 | 1.68E-12 | 2.05E-11 |
| CTNNA2 | 4.963234 | 2.378226 | -1.06139 | 3.38E-05 | 9.73E-05 |
| MLPH | 24.6502 | 11.83705 | -1.05829 | 2.52E-18 | 1.07E-16 |
| ADAM28 | 6.560262 | 3.151471 | -1.05773 | 9.08E-19 | 4.40E-17 |
| SPINK4 | 1.098703 | 0.52924 | -1.05381 | 1.52E-15 | 3.43E-14 |
| DEFB1 | 102.7587 | 49.50068 | -1.05374 | 1.16E-05 | 3.66E-05 |
| KCNJ15 | 1.322807 | 0.637229 | -1.05372 | 1.14E-05 | 3.58E-05 |
| TLR4 | 2.293692 | 1.105473 | -1.05301 | 1.74E-13 | 2.60E-12 |
| CCDC176 | 3.609939 | 1.7417 | -1.05148 | 2.92E-12 | 3.38E-11 |
| FAM13C | 0.459774 | 0.222217 | -1.04896 | 2.26E-12 | 2.68E-11 |
| EFCAB12 | 1.61104 | 0.779743 | -1.04692 | 7.19E-05 | 0.000193 |
| MYLK3 | 0.580832 | 0.281215 | -1.04645 | 0.003859 | 0.007185 |
| FAM189A1 | 1.50546 | 0.72913 | -1.04596 | 3.50E-10 | 2.67E-09 |
| SCUBE2 | 4.486081 | 2.174326 | -1.04489 | 0.017821 | 0.02835 |
| NEK5 | 1.385262 | 0.671733 | -1.0442 | 3.15E-08 | 1.64E-07 |
| LIPM | 0.448785 | 0.218096 | -1.04106 | 7.47E-15 | 1.47E-13 |
| ADAMTS18 | 0.740061 | 0.360041 | -1.03948 | 3.47E-07 | 1.48E-06 |
| TCF7 | 5.203073 | 2.536088 | -1.03676 | 2.70E-15 | 5.79E-14 |
| IGSF5 | 0.282664 | 0.138313 | -1.03115 | 1.27E-08 | 7.08E-08 |
| CEACAM1 | 9.997814 | 4.90511 | -1.02733 | 3.58E-19 | 2.01E-17 |
| VWA5B2 | 3.742133 | 1.836952 | -1.02655 | 6.58E-07 | 2.63E-06 |
| SMAD9 | 3.577899 | 1.756504 | -1.02641 | 1.62E-13 | 2.45E-12 |
| ELP3 | 29.24298 | 14.35955 | -1.02608 | 3.06E-19 | 1.75E-17 |
| TTC9 | 8.903556 | 4.377984 | -1.02411 | 1.55E-22 | 1.93E-20 |
| CPN1 | 0.275252 | 0.135395 | -1.02358 | 0.008208 | 0.014177 |
| PRRX1 | 9.652057 | 4.75132 | -1.02251 | 4.92E-14 | 8.30E-13 |
| PLA2G10 | 1.877688 | 0.925828 | -1.02014 | 2.13E-10 | 1.69E-09 |
| NPDC1 | 81.69366 | 40.28953 | -1.01982 | 7.11E-26 | 2.30E-23 |
| KLHL6 | 0.863755 | 0.426219 | -1.01903 | 2.29E-05 | 6.80E-05 |
| DUOX1 | 4.002899 | 1.976603 | -1.01802 | 1.05E-06 | 4.07E-06 |
| FCRL2 | 0.401113 | 0.198071 | -1.01799 | 7.49E-08 | 3.63E-07 |
| VIM | 430.5773 | 212.6205 | -1.01799 | 7.45E-21 | 5.93E-19 |
| PHYHIPL | 3.368286 | 1.669048 | -1.01299 | 4.64E-07 | 1.92E-06 |
| ALDH1A3 | 1.620729 | 0.803268 | -1.01269 | 2.76E-20 | 1.95E-18 |
| TSPAN5 | 3.150628 | 1.564007 | -1.01039 | 1.41E-17 | 4.89E-16 |
| CHST6 | 7.317129 | 3.632792 | -1.0102 | 2.24E-10 | 1.77E-09 |
| CEACAM21 | 5.335007 | 2.652446 | -1.00817 | 8.00E-08 | 3.86E-07 |
| ADRA2A | 6.362203 | 3.16558 | -1.00706 | 1.03E-12 | 1.32E-11 |
| CUBN | 0.561045 | 0.279566 | -1.00493 | 1.18E-16 | 3.44E-15 |
| MUC13 | 9.848527 | 4.908752 | -1.00455 | 3.02E-15 | 6.41E-14 |
| TP63 | 0.61796 | 0.30827 | -1.00332 | 2.32E-05 | 6.87E-05 |
| FEZF1 | 0.249093 | 0.124323 | -1.0026 | 0.000722 | 0.001583 |
| COL8A2 | 2.046612 | 4.093331 | 1.000038 | 1.97E-09 | 1.29E-08 |
| SEMA6D | 0.346109 | 0.692734 | 1.001076 | 2.68E-07 | 1.17E-06 |
| XKR9 | 0.205876 | 0.412088 | 1.001173 | 4.06E-05 | 0.000115 |
| CCNI2 | 0.161305 | 0.323002 | 1.001751 | 5.13E-09 | 3.11E-08 |
| ALX1 | 0.271253 | 0.543398 | 1.00237 | 1.64E-08 | 9.00E-08 |
| LGI2 | 0.550735 | 1.103736 | 1.002964 | 1.01E-11 | 1.06E-10 |
| RIMS3 | 0.354902 | 0.711682 | 1.003812 | 3.50E-13 | 4.96E-12 |
| ZNF829 | 0.423606 | 0.849478 | 1.003855 | 6.79E-19 | 3.46E-17 |
| TPX2 | 18.98 | 38.11626 | 1.005927 | 1.81E-31 | 3.52E-28 |
| FAM131C | 0.524374 | 1.054149 | 1.007411 | 1.46E-13 | 2.22E-12 |
| ZFR2 | 0.152704 | 0.307156 | 1.008236 | 0.000381 | 0.000884 |
| ZNF114 | 0.958317 | 1.927649 | 1.008268 | 6.95E-10 | 4.94E-09 |
| SLC3A1 | 0.971631 | 1.954898 | 1.008613 | 0.01154 | 0.019225 |
| TLL2 | 0.255726 | 0.515264 | 1.010717 | 0.038231 | 0.056364 |
| ARMCX1 | 3.67055 | 7.401197 | 1.011763 | 8.61E-13 | 1.12E-11 |
| EDARADD | 0.557069 | 1.123331 | 1.011855 | 8.03E-09 | 4.67E-08 |
| GLOD5 | 0.494824 | 0.9992 | 1.01386 | 2.83E-06 | 1.00E-05 |
| URI1 | 11.15695 | 22.53083 | 1.013958 | 4.40E-21 | 3.74E-19 |
| SLC17A9 | 1.68208 | 3.399877 | 1.015236 | 1.79E-08 | 9.77E-08 |
| SEC14L4 | 0.347207 | 0.701887 | 1.015443 | 6.26E-07 | 2.52E-06 |
| SYN1 | 0.472825 | 0.956897 | 1.017058 | 4.46E-10 | 3.30E-09 |
| HMGA2 | 1.576237 | 3.191386 | 1.017699 | 4.18E-06 | 1.44E-05 |
| LY6K | 0.793581 | 1.610297 | 1.020878 | 6.69E-09 | 3.94E-08 |
| CLDN1 | 14.23252 | 28.88152 | 1.020955 | 5.24E-07 | 2.15E-06 |
| ENSG00000233757 | 0.152178 | 0.308934 | 1.021537 | 1.59E-08 | 8.76E-08 |
| ZNF572 | 0.746891 | 1.517166 | 1.022409 | 1.98E-17 | 6.58E-16 |
| WSCD1 | 0.330409 | 0.671545 | 1.023233 | 0.000854 | 0.001845 |
| NHLRC1 | 1.228117 | 2.496683 | 1.023564 | 7.81E-18 | 2.88E-16 |
| SLC38A1 | 10.35662 | 21.06352 | 1.024193 | 2.66E-21 | 2.47E-19 |
| ZFPM2 | 0.184517 | 0.375754 | 1.026035 | 9.82E-11 | 8.36E-10 |
| C7orf61 | 0.531909 | 1.084123 | 1.027278 | 2.33E-11 | 2.24E-10 |
| NXPH4 | 4.743905 | 9.670401 | 1.0275 | 7.51E-13 | 9.88E-12 |
| KCNIP3 | 0.80476 | 1.642013 | 1.028836 | 5.00E-16 | 1.27E-14 |
| MUC20 | 5.848151 | 11.93323 | 1.028932 | 9.33E-11 | 7.98E-10 |
| NEURL1 | 0.489628 | 0.999304 | 1.029238 | 6.98E-05 | 0.000188 |
| NTF3 | 0.336218 | 0.686357 | 1.029561 | 0.000203 | 0.000498 |
| NDC80 | 4.265927 | 8.713555 | 1.030402 | 1.97E-25 | 5.37E-23 |
| MPV17L | 0.709269 | 1.448858 | 1.03051 | 2.28E-10 | 1.80E-09 |
| ACOXL | 0.185405 | 0.379102 | 1.031909 | 4.74E-17 | 1.47E-15 |
| CRIP3 | 0.319738 | 0.653891 | 1.032159 | 2.52E-10 | 1.97E-09 |
| RNF165 | 0.162659 | 0.332893 | 1.033211 | 7.55E-13 | 9.90E-12 |
| LRRC3 | 0.570365 | 1.168192 | 1.03432 | 9.97E-22 | 1.05E-19 |
| TTK | 3.234321 | 6.626158 | 1.03471 | 5.91E-28 | 3.14E-25 |
| EPHB6 | 1.803645 | 3.697735 | 1.035727 | 8.53E-09 | 4.94E-08 |
| CACNA1A | 0.350585 | 0.718779 | 1.035785 | 1.97E-08 | 1.07E-07 |
| PLCH2 | 0.351659 | 0.721175 | 1.036173 | 0.002394 | 0.004677 |
| FGFR3 | 5.020884 | 10.30291 | 1.037039 | 5.23E-06 | 1.76E-05 |
| KCNE2 | 0.239027 | 0.490563 | 1.037264 | 1.60E-17 | 5.48E-16 |
| KCNMB3 | 0.154156 | 0.317077 | 1.040439 | 2.07E-20 | 1.50E-18 |
| FTCD | 0.311784 | 0.641427 | 1.04074 | 4.20E-07 | 1.76E-06 |
| IFIT3 | 11.21718 | 23.11253 | 1.042965 | 9.64E-13 | 1.24E-11 |
| SPSB4 | 0.266245 | 0.549785 | 1.046115 | 9.80E-07 | 3.80E-06 |
| TMEM52B | 0.149794 | 0.309418 | 1.04658 | 0.009314 | 0.015873 |
| GALNT13 | 0.144779 | 0.299161 | 1.047067 | 0.000184 | 0.000457 |
| DSEL | 0.322613 | 0.667448 | 1.048852 | 9.25E-07 | 3.61E-06 |
| TMEM38A | 3.40092 | 7.039448 | 1.049537 | 4.12E-20 | 2.87E-18 |
| KCP | 1.267763 | 2.632665 | 1.054239 | 3.05E-10 | 2.34E-09 |
| HIST1H3B | 2.732098 | 5.679749 | 1.055818 | 5.96E-07 | 2.41E-06 |
| SLC16A10 | 0.362054 | 0.753362 | 1.057138 | 2.89E-12 | 3.34E-11 |
| TCF15 | 0.803336 | 1.673243 | 1.058572 | 3.78E-10 | 2.85E-09 |
| GSTM2 | 1.085933 | 2.263265 | 1.05947 | 1.11E-15 | 2.59E-14 |
| SYNGR3 | 0.890534 | 1.857932 | 1.060955 | 1.18E-15 | 2.73E-14 |
| ZNF662 | 0.496907 | 1.038569 | 1.063548 | 4.18E-11 | 3.81E-10 |
| PELI2 | 1.455104 | 3.045656 | 1.06563 | 6.95E-14 | 1.13E-12 |
| CHODL | 0.935146 | 1.957484 | 1.065737 | 1.03E-12 | 1.32E-11 |
| WISP3 | 0.376601 | 0.788634 | 1.066319 | 1.03E-06 | 3.97E-06 |
| HOGA1 | 0.468963 | 0.982726 | 1.067316 | 0.00375 | 0.007004 |
| STX1B | 0.149539 | 0.313766 | 1.069162 | 1.76E-06 | 6.52E-06 |
| DUSP26 | 0.328064 | 0.688424 | 1.069321 | 0.020331 | 0.031977 |
| PXDC1 | 6.395608 | 13.426 | 1.069876 | 8.93E-22 | 9.51E-20 |
| NKX2-8 | 0.224021 | 0.470577 | 1.070794 | 5.57E-14 | 9.28E-13 |
| SELV | 0.273489 | 0.575596 | 1.073575 | 6.12E-07 | 2.47E-06 |
| IRS2 | 1.888119 | 3.975541 | 1.074202 | 2.06E-09 | 1.35E-08 |
| SORCS2 | 0.706879 | 1.488399 | 1.074226 | 0.002793 | 0.005372 |
| FBXL16 | 1.56051 | 3.287342 | 1.074904 | 6.31E-17 | 1.91E-15 |
| IQCA1 | 0.736901 | 1.552849 | 1.075374 | 2.12E-08 | 1.14E-07 |
| RAB39B | 0.451483 | 0.953166 | 1.078055 | 3.95E-15 | 8.22E-14 |
| RBP7 | 3.002622 | 6.340576 | 1.078391 | 0.014513 | 0.023572 |
| BARX2 | 0.592813 | 1.252477 | 1.079135 | 3.55E-07 | 1.51E-06 |
| LGALS7 | 0.271421 | 0.574369 | 1.081444 | 3.36E-05 | 9.68E-05 |
| MIOX | 0.499413 | 1.059615 | 1.085237 | 0.00164 | 0.003326 |
| TMIE | 0.422267 | 0.89607 | 1.085458 | 9.84E-16 | 2.34E-14 |
| PRKCI | 7.62624 | 16.18553 | 1.08566 | 3.56E-24 | 7.04E-22 |
| ITGA7 | 1.658341 | 3.520882 | 1.086197 | 1.68E-12 | 2.05E-11 |
| TRIM58 | 0.200961 | 0.427255 | 1.088184 | 2.36E-05 | 6.98E-05 |
| IQCJ-SCHIP1 | 0.205597 | 0.437371 | 1.089036 | 1.32E-11 | 1.34E-10 |
| APOBEC3A | 0.333612 | 0.710709 | 1.091087 | 1.49E-07 | 6.81E-07 |
| ADCY9 | 1.046008 | 2.229691 | 1.09195 | 6.12E-20 | 4.01E-18 |
| GLIS2 | 5.026478 | 10.71778 | 1.092386 | 1.03E-21 | 1.06E-19 |
| FAM90A1 | 0.766406 | 1.635208 | 1.093294 | 3.03E-16 | 8.14E-15 |
| DTNA | 0.321578 | 0.686259 | 1.093582 | 3.44E-08 | 1.77E-07 |
| SMTNL1 | 0.216357 | 0.462135 | 1.094903 | 3.97E-11 | 3.65E-10 |
| FOXG1 | 1.038424 | 2.219307 | 1.095713 | 0.001159 | 0.002425 |
| IGSF23 | 0.775514 | 1.659671 | 1.097672 | 1.22E-10 | 1.02E-09 |
| MAGEH1 | 4.629989 | 9.921106 | 1.099492 | 3.60E-15 | 7.54E-14 |
| CDKN2C | 3.660599 | 7.852766 | 1.101121 | 2.09E-10 | 1.67E-09 |
| CR2 | 0.434686 | 0.932706 | 1.101449 | 5.06E-09 | 3.07E-08 |
| ST6GALNAC5 | 0.571642 | 1.228242 | 1.103412 | 0.007139 | 0.012491 |
| EPHX4 | 0.619328 | 1.330999 | 1.103733 | 5.32E-12 | 5.83E-11 |
| PHOSPHO1 | 0.326474 | 0.701796 | 1.104083 | 6.26E-09 | 3.71E-08 |
| MATN4 | 0.304343 | 0.6545 | 1.104694 | 2.25E-08 | 1.20E-07 |
| CRYAB | 11.76485 | 25.31685 | 1.105615 | 4.62E-08 | 2.32E-07 |
| MB21D2 | 1.447259 | 3.12298 | 1.109601 | 1.37E-10 | 1.13E-09 |
| BEX2 | 10.01548 | 21.62265 | 1.110312 | 3.05E-22 | 3.58E-20 |
| CACNA1B | 0.154283 | 0.333145 | 1.110571 | 1.30E-08 | 7.24E-08 |
| KLC3 | 1.124763 | 2.435247 | 1.114447 | 3.02E-15 | 6.41E-14 |
| FXYD6 | 2.773651 | 6.007527 | 1.114985 | 1.47E-15 | 3.32E-14 |
| SPEG | 0.577027 | 1.250677 | 1.115998 | 3.42E-12 | 3.91E-11 |
| CPXM2 | 3.889163 | 8.435593 | 1.11703 | 4.87E-05 | 0.000136 |
| AC005480.1 | 0.210438 | 0.45645 | 1.117064 | 0.007758 | 0.013482 |
| EXOC3L4 | 0.46475 | 1.008684 | 1.117946 | 1.00E-08 | 5.71E-08 |
| KCNJ4 | 0.515733 | 1.119547 | 1.11822 | 3.94E-08 | 2.00E-07 |
| RUNDC3A | 0.342744 | 0.745016 | 1.120142 | 1.35E-08 | 7.54E-08 |
| AMN | 1.574578 | 3.424576 | 1.120961 | 8.78E-09 | 5.07E-08 |
| PIP5KL1 | 0.531797 | 1.158369 | 1.123147 | 7.66E-21 | 6.03E-19 |
| FABP3 | 10.7328 | 23.37867 | 1.123166 | 2.85E-13 | 4.11E-12 |
| SPIRE1 | 1.227211 | 2.676195 | 1.1248 | 2.18E-18 | 9.59E-17 |
| XKR6 | 0.145473 | 0.317545 | 1.126208 | 7.23E-11 | 6.30E-10 |
| GFOD1 | 0.858512 | 1.874177 | 1.126347 | 1.44E-17 | 4.94E-16 |
| UQCRFS1 | 15.38557 | 33.63288 | 1.128294 | 2.74E-16 | 7.50E-15 |
| PNMA2 | 0.256653 | 0.562194 | 1.131246 | 5.54E-05 | 0.000153 |
| CACNG4 | 2.854334 | 6.25745 | 1.132421 | 5.03E-09 | 3.06E-08 |
| HDX | 0.204114 | 0.447525 | 1.132592 | 3.07E-09 | 1.94E-08 |
| TMEM176A | 9.572056 | 21.01236 | 1.134338 | 3.22E-06 | 1.13E-05 |
| ASTL | 0.17009 | 0.373439 | 1.13457 | 4.51E-09 | 2.77E-08 |
| UBE2U | 0.137208 | 0.301545 | 1.136006 | 1.32E-05 | 4.10E-05 |
| MAST1 | 0.755127 | 1.660042 | 1.136428 | 5.86E-18 | 2.25E-16 |
| C19orf57 | 1.026922 | 2.259276 | 1.137534 | 4.33E-19 | 2.34E-17 |
| SLC12A5 | 0.282362 | 0.621401 | 1.137979 | 1.09E-15 | 2.54E-14 |
| RPL39L | 11.37993 | 25.0702 | 1.139482 | 4.24E-20 | 2.93E-18 |
| THEMIS2 | 5.216793 | 11.52248 | 1.143215 | 5.41E-22 | 5.94E-20 |
| APLP1 | 4.167877 | 9.207549 | 1.143504 | 4.94E-17 | 1.53E-15 |
| GCK | 0.244966 | 0.541226 | 1.143646 | 2.56E-09 | 1.64E-08 |
| OAS1 | 6.692564 | 14.80664 | 1.145613 | 2.13E-15 | 4.65E-14 |
| CT45A10 | 0.323104 | 0.71554 | 1.147034 | 2.49E-09 | 1.60E-08 |
| COL9A3 | 2.601711 | 5.773718 | 1.15004 | 2.93E-07 | 1.27E-06 |
| SOSTDC1 | 1.697545 | 3.770045 | 1.151132 | 0.047654 | 0.068471 |
| SNAP25 | 0.452941 | 1.007203 | 1.152959 | 1.80E-12 | 2.18E-11 |
| FAM107A | 7.081816 | 15.7591 | 1.153994 | 1.57E-14 | 2.91E-13 |
| KCNQ3 | 0.241264 | 0.537778 | 1.156397 | 0.000192 | 0.000474 |
| RAB42 | 1.430647 | 3.195096 | 1.159191 | 7.32E-09 | 4.29E-08 |
| IFI6 | 404.0709 | 902.5 | 1.159318 | 3.15E-11 | 2.96E-10 |
| LRRC14B | 0.15943 | 0.356173 | 1.159652 | 7.77E-12 | 8.27E-11 |
| STAC | 0.211374 | 0.472231 | 1.159694 | 1.26E-10 | 1.05E-09 |
| TNFAIP6 | 0.797722 | 1.784628 | 1.161665 | 4.26E-05 | 0.00012 |
| RASL10B | 1.230866 | 2.754963 | 1.162359 | 7.27E-14 | 1.18E-12 |
| ATP2B2 | 0.692662 | 1.552947 | 1.164786 | 1.70E-07 | 7.69E-07 |
| CCDC150 | 0.397504 | 0.893384 | 1.168311 | 2.20E-26 | 8.11E-24 |
| POP4 | 4.71031 | 10.58999 | 1.168807 | 1.02E-25 | 3.16E-23 |
| POU2F3 | 0.244454 | 0.549615 | 1.168858 | 4.02E-16 | 1.05E-14 |
| DSCAML1 | 0.154727 | 0.348276 | 1.170504 | 1.63E-12 | 2.00E-11 |
| SCEL | 1.038713 | 2.345611 | 1.175167 | 2.29E-06 | 8.31E-06 |
| GPR156 | 0.241228 | 0.544977 | 1.175796 | 2.15E-11 | 2.09E-10 |
| ADAM33 | 1.33486 | 3.016248 | 1.176066 | 6.64E-07 | 2.65E-06 |
| KCNJ12 | 0.792702 | 1.791279 | 1.176139 | 1.03E-12 | 1.32E-11 |
| STEAP1B | 0.172236 | 0.390354 | 1.180399 | 4.84E-15 | 9.89E-14 |
| NAT8L | 4.187433 | 9.498851 | 1.181687 | 1.19E-11 | 1.23E-10 |
| KLHL30 | 0.485657 | 1.101901 | 1.181985 | 4.90E-16 | 1.25E-14 |
| PDE6G | 1.015357 | 2.304634 | 1.18255 | 8.36E-10 | 5.84E-09 |
| LRRN1 | 1.899022 | 4.328055 | 1.188463 | 1.33E-05 | 4.13E-05 |
| ELOVL4 | 0.478196 | 1.090807 | 1.189722 | 7.36E-09 | 4.31E-08 |
| LGALS7B | 2.468237 | 5.633796 | 1.190626 | 3.75E-10 | 2.83E-09 |
| KRT7 | 67.04697 | 153.4226 | 1.194267 | 1.07E-20 | 8.10E-19 |
| ASS1 | 39.16622 | 89.9875 | 1.200115 | 5.30E-16 | 1.34E-14 |
| HIST1H2BO | 1.44076 | 3.312707 | 1.201181 | 8.86E-12 | 9.37E-11 |
| EPHB1 | 0.333269 | 0.767425 | 1.20334 | 1.44E-10 | 1.18E-09 |
| TNFSF10 | 24.73883 | 57.3655 | 1.213406 | 2.47E-12 | 2.91E-11 |
| KCNH2 | 1.451419 | 3.365808 | 1.213489 | 1.83E-16 | 5.15E-15 |
| CDH16 | 4.213782 | 9.785083 | 1.215468 | 0.00983 | 0.016667 |
| MAP7D2 | 0.603403 | 1.40223 | 1.216528 | 1.12E-07 | 5.26E-07 |
| GPHA2 | 0.335928 | 0.781896 | 1.218823 | 6.36E-07 | 2.56E-06 |
| SNX32 | 0.213194 | 0.497936 | 1.223795 | 6.01E-16 | 1.50E-14 |
| BST2 | 227.7566 | 532.6115 | 1.225591 | 4.08E-15 | 8.44E-14 |
| IFI44 | 11.11301 | 25.99162 | 1.225797 | 3.37E-15 | 7.10E-14 |
| OR2W3 | 0.212391 | 0.497825 | 1.228914 | 0.006099 | 0.010866 |
| SUSD3 | 4.077644 | 9.560543 | 1.229357 | 3.56E-12 | 4.05E-11 |
| LECT1 | 0.201566 | 0.472866 | 1.230179 | 0.000111 | 0.000286 |
| GSPT2 | 0.845477 | 1.985028 | 1.231322 | 0.006076 | 0.010826 |
| C8orf88 | 0.338946 | 0.796404 | 1.232447 | 1.61E-05 | 4.92E-05 |
| CDKL2 | 0.205672 | 0.483616 | 1.233515 | 2.94E-14 | 5.14E-13 |
| RASGEF1A | 1.178426 | 2.773759 | 1.234982 | 3.92E-16 | 1.03E-14 |
| GAL | 1.720973 | 4.054483 | 1.236293 | 8.69E-09 | 5.03E-08 |
| PCDHB6 | 0.350719 | 0.826683 | 1.237017 | 0.000775 | 0.001689 |
| GPX3 | 69.44893 | 163.7497 | 1.237467 | 4.58E-07 | 1.90E-06 |
| KIF5A | 0.295837 | 0.700112 | 1.242785 | 2.98E-08 | 1.55E-07 |
| KCNS1 | 0.315106 | 0.746401 | 1.244111 | 1.55E-19 | 9.56E-18 |
| SYNDIG1 | 1.07041 | 2.539197 | 1.246209 | 3.30E-06 | 1.16E-05 |
| ITM2C | 105.2579 | 249.7783 | 1.24672 | 3.60E-15 | 7.54E-14 |
| ZNF835 | 0.184546 | 0.43811 | 1.247313 | 0.002991 | 0.005716 |
| CHP2 | 0.257486 | 0.61198 | 1.248991 | 0.017003 | 0.027217 |
| MSLN | 54.52134 | 129.665 | 1.249896 | 9.32E-06 | 2.99E-05 |
| CARD11 | 1.259281 | 2.994942 | 1.249927 | 1.46E-09 | 9.76E-09 |
| CPVL | 4.235611 | 10.07384 | 1.249972 | 4.99E-23 | 7.08E-21 |
| KIF1A | 6.080697 | 14.48107 | 1.251859 | 6.13E-16 | 1.52E-14 |
| MAP10 | 0.145794 | 0.347229 | 1.25196 | 0.024985 | 0.038406 |
| HOXA2 | 0.562409 | 1.34137 | 1.254017 | 2.91E-08 | 1.52E-07 |
| LMO1 | 0.701972 | 1.677308 | 1.256664 | 9.57E-21 | 7.41E-19 |
| BTBD17 | 0.347315 | 0.830724 | 1.258122 | 2.82E-14 | 4.94E-13 |
| BEND6 | 0.168604 | 0.40343 | 1.258683 | 8.16E-11 | 7.04E-10 |
| FAM110B | 1.244462 | 2.996613 | 1.267811 | 4.03E-17 | 1.26E-15 |
| HPSE | 0.75699 | 1.824362 | 1.269046 | 1.35E-13 | 2.08E-12 |
| HIST1H3G | 0.558955 | 1.347383 | 1.269356 | 1.02E-05 | 3.26E-05 |
| IFIT2 | 3.301637 | 7.965665 | 1.270613 | 8.49E-14 | 1.36E-12 |
| COL25A1 | 0.495593 | 1.197063 | 1.272271 | 2.05E-08 | 1.10E-07 |
| PRAP1 | 2.351853 | 5.686126 | 1.273648 | 4.36E-10 | 3.24E-09 |
| UPK1A | 0.408161 | 0.989301 | 1.277271 | 1.80E-07 | 8.09E-07 |
| OASL | 2.552955 | 6.191066 | 1.27802 | 1.77E-17 | 5.93E-16 |
| CHL1 | 0.957132 | 2.32347 | 1.279491 | 6.91E-06 | 2.27E-05 |
| ART5 | 0.500736 | 1.215576 | 1.279519 | 4.09E-13 | 5.72E-12 |
| TGM7 | 1.409014 | 3.42769 | 1.282551 | 0.004105 | 0.007595 |
| PTGS1 | 11.29746 | 27.50646 | 1.283771 | 1.17E-12 | 1.47E-11 |
| ATP1A3 | 0.222831 | 0.542734 | 1.284298 | 4.68E-06 | 1.59E-05 |
| GPR19 | 0.224648 | 0.548171 | 1.28696 | 4.37E-23 | 6.40E-21 |
| MYOM3 | 0.15729 | 0.383896 | 1.287292 | 0.012186 | 0.020161 |
| ZFP92 | 0.569016 | 1.389441 | 1.287962 | 1.37E-11 | 1.38E-10 |
| COX4I2 | 2.070773 | 5.068766 | 1.291465 | 0.000231 | 0.00056 |
| CHST13 | 0.382551 | 0.936851 | 1.292167 | 9.81E-10 | 6.75E-09 |
| NPTX2 | 4.40664 | 10.79756 | 1.292954 | 9.26E-09 | 5.32E-08 |
| FAM132A | 1.097232 | 2.691271 | 1.294419 | 3.38E-10 | 2.58E-09 |
| LRFN1 | 1.235105 | 3.030656 | 1.294996 | 1.36E-21 | 1.35E-19 |
| PRKCG | 0.58733 | 1.443765 | 1.297594 | 7.36E-05 | 0.000198 |
| NOS1 | 0.134005 | 0.329708 | 1.298908 | 0.002732 | 0.005266 |
| GLDN | 0.240621 | 0.592574 | 1.300232 | 4.70E-09 | 2.87E-08 |
| EN1 | 0.211249 | 0.520391 | 1.300651 | 0.000391 | 0.000905 |
| RGN | 0.461986 | 1.139345 | 1.302285 | 0.000149 | 0.000375 |
| CLDN11 | 0.382843 | 0.945762 | 1.304725 | 0.000174 | 0.000434 |
| COL11A2 | 0.56394 | 1.394563 | 1.3062 | 6.59E-12 | 7.11E-11 |
| TMEM130 | 0.466652 | 1.154059 | 1.306297 | 3.23E-05 | 9.34E-05 |
| PKIA | 0.60084 | 1.497554 | 1.317554 | 4.01E-12 | 4.51E-11 |
| LYPD6B | 2.670601 | 6.662097 | 1.318812 | 1.26E-12 | 1.58E-11 |
| ZYG11A | 0.361898 | 0.903474 | 1.3199 | 1.48E-16 | 4.27E-15 |
| TMEM221 | 0.60986 | 1.523557 | 1.320895 | 3.95E-10 | 2.96E-09 |
| BMP7 | 5.960762 | 14.90655 | 1.322378 | 1.32E-13 | 2.03E-12 |
| S100A5 | 0.590804 | 1.478252 | 1.323142 | 1.98E-20 | 1.45E-18 |
| CHAD | 0.282239 | 0.706296 | 1.323354 | 1.55E-05 | 4.76E-05 |
| CLCN4 | 0.713165 | 1.787649 | 1.325756 | 1.16E-23 | 2.08E-21 |
| TRIM43 | 0.130721 | 0.327931 | 1.326903 | 6.89E-05 | 0.000186 |
| CPS1 | 0.265657 | 0.668457 | 1.33127 | 4.85E-09 | 2.96E-08 |
| PRSS21 | 3.818192 | 9.623462 | 1.333666 | 1.57E-08 | 8.67E-08 |
| CEND1 | 0.277072 | 0.699175 | 1.335394 | 8.29E-05 | 0.00022 |
| C19orf33 | 41.235 | 104.0975 | 1.335995 | 7.40E-14 | 1.20E-12 |
| TMEM171 | 0.30922 | 0.780757 | 1.33624 | 5.50E-06 | 1.84E-05 |
| RBP2 | 0.358074 | 0.906611 | 1.340224 | 3.43E-09 | 2.15E-08 |
| THPO | 0.369139 | 0.935083 | 1.340928 | 0.000168 | 0.000419 |
| TMTC1 | 1.185825 | 3.003876 | 1.340934 | 1.69E-11 | 1.68E-10 |
| COL23A1 | 3.045348 | 7.714473 | 1.34096 | 4.95E-12 | 5.46E-11 |
| MX1 | 13.0933 | 33.20584 | 1.342609 | 1.45E-14 | 2.71E-13 |
| CAMKV | 0.133512 | 0.338644 | 1.342805 | 2.34E-06 | 8.47E-06 |
| SLC39A5 | 0.500713 | 1.270027 | 1.342805 | 4.94E-11 | 4.43E-10 |
| ERP27 | 3.666171 | 9.300193 | 1.342987 | 0.000402 | 0.00093 |
| BIRC7 | 0.778359 | 1.978158 | 1.34565 | 4.76E-07 | 1.97E-06 |
| LRRN2 | 6.442653 | 16.39435 | 1.347472 | 1.63E-15 | 3.64E-14 |
| RBM11 | 0.257703 | 0.656349 | 1.348752 | 2.40E-17 | 7.80E-16 |
| SYCP2 | 0.203289 | 0.521331 | 1.358668 | 2.86E-09 | 1.81E-08 |
| WNT10A | 2.172867 | 5.577933 | 1.360131 | 1.44E-07 | 6.59E-07 |
| SSC4D | 1.6538 | 4.25592 | 1.363686 | 2.98E-18 | 1.24E-16 |
| MC4R | 0.16295 | 0.41981 | 1.365302 | 0.014522 | 0.023585 |
| NPFFR1 | 0.225617 | 0.58146 | 1.365802 | 0.000139 | 0.000352 |
| ELOVL2 | 0.239935 | 0.619737 | 1.369013 | 7.93E-10 | 5.57E-09 |
| LAMP3 | 1.997256 | 5.161144 | 1.369672 | 4.48E-18 | 1.80E-16 |
| RPS6KA6 | 0.167148 | 0.433676 | 1.37549 | 1.88E-06 | 6.91E-06 |
| FOXO6 | 1.954825 | 5.07431 | 1.376172 | 8.49E-18 | 3.11E-16 |
| IQSEC3 | 0.281997 | 0.732035 | 1.37623 | 5.70E-10 | 4.12E-09 |
| TNNT3 | 0.46028 | 1.195327 | 1.376823 | 0.020895 | 0.032744 |
| IFI44L | 2.565007 | 6.669374 | 1.378589 | 6.30E-15 | 1.26E-13 |
| KRTAP2-3 | 0.580557 | 1.510606 | 1.379618 | 2.18E-06 | 7.94E-06 |
| THRB | 0.641767 | 1.674238 | 1.383384 | 6.32E-21 | 5.12E-19 |
| CPNE8 | 0.892361 | 2.328943 | 1.383976 | 3.59E-10 | 2.72E-09 |
| RSAD2 | 2.298314 | 6.008792 | 1.386499 | 2.41E-18 | 1.03E-16 |
| EPHB2 | 1.671978 | 4.398806 | 1.395556 | 3.68E-26 | 1.30E-23 |
| CILP2 | 1.395316 | 3.671885 | 1.395929 | 1.68E-14 | 3.11E-13 |
| UCMA | 0.471272 | 1.24083 | 1.396672 | 0.009924 | 0.01681 |
| SLC8A1 | 0.419895 | 1.110543 | 1.403164 | 6.15E-16 | 1.52E-14 |
| ADAMTS16 | 0.437725 | 1.159069 | 1.404871 | 4.38E-05 | 0.000124 |
| LRFN5 | 0.202011 | 0.534953 | 1.404975 | 0.001268 | 0.002628 |
| MLF1 | 4.398925 | 11.65822 | 1.406124 | 1.69E-23 | 2.80E-21 |
| NUDT11 | 0.503018 | 1.333582 | 1.406624 | 0.00106 | 0.002241 |
| RASSF10 | 1.578953 | 4.196772 | 1.410312 | 2.20E-09 | 1.43E-08 |
| LRP2 | 0.502747 | 1.336556 | 1.410616 | 1.02E-05 | 3.25E-05 |
| S100A1 | 9.800296 | 26.06606 | 1.411275 | 2.81E-16 | 7.64E-15 |
| TRPM5 | 0.189354 | 0.506812 | 1.420369 | 1.89E-05 | 5.70E-05 |
| NPTXR | 3.466606 | 9.282173 | 1.420939 | 3.16E-18 | 1.31E-16 |
| SLC19A3 | 0.363393 | 0.97546 | 1.42455 | 1.09E-06 | 4.19E-06 |
| GPR173 | 1.479475 | 3.975085 | 1.4259 | 4.84E-22 | 5.41E-20 |
| MACROD2 | 0.867118 | 2.333069 | 1.427928 | 5.92E-10 | 4.27E-09 |
| KHDC1 | 0.41714 | 1.122746 | 1.428428 | 1.31E-25 | 3.71E-23 |
| PPAPDC1A | 1.256533 | 3.38469 | 1.429576 | 7.26E-11 | 6.32E-10 |
| SPON1 | 24.22062 | 65.41349 | 1.433352 | 1.02E-11 | 1.07E-10 |
| SOHLH1 | 0.418265 | 1.130718 | 1.43475 | 2.37E-06 | 8.59E-06 |
| SALL4 | 0.516908 | 1.397456 | 1.434823 | 1.16E-10 | 9.74E-10 |
| DUSP9 | 0.701978 | 1.899026 | 1.435763 | 2.14E-17 | 7.01E-16 |
| ONECUT2 | 0.196245 | 0.532825 | 1.441009 | 0.000222 | 0.000541 |
| IGF2BP2 | 5.543156 | 15.09131 | 1.444939 | 1.29E-16 | 3.74E-15 |
| ADTRP | 0.189813 | 0.516994 | 1.445568 | 0.000216 | 0.000526 |
| ESPNL | 0.287158 | 0.78324 | 1.44761 | 4.47E-12 | 4.97E-11 |
| CD22 | 0.574071 | 1.568767 | 1.45033 | 5.21E-08 | 2.59E-07 |
| TENM1 | 0.202395 | 0.553524 | 1.45147 | 1.17E-10 | 9.76E-10 |
| FSD1 | 0.723668 | 1.981303 | 1.45305 | 3.18E-15 | 6.73E-14 |
| ADAMTS15 | 4.187357 | 11.46508 | 1.453134 | 2.23E-14 | 4.01E-13 |
| PIPOX | 0.393969 | 1.079255 | 1.453884 | 5.37E-07 | 2.20E-06 |
| ARHGAP29 | 1.680592 | 4.614741 | 1.457281 | 3.82E-21 | 3.36E-19 |
| ZNF492 | 0.18588 | 0.510429 | 1.45734 | 8.31E-13 | 1.08E-11 |
| WNK3 | 0.202653 | 0.558701 | 1.463066 | 1.97E-19 | 1.17E-17 |
| ADRA2B | 0.280056 | 0.773019 | 1.46479 | 1.77E-12 | 2.14E-11 |
| DMRT3 | 0.605428 | 1.678221 | 1.470906 | 9.43E-13 | 1.21E-11 |
| HORMAD1 | 0.292112 | 0.811516 | 1.474099 | 0.002361 | 0.00462 |
| AVPR2 | 0.483048 | 1.343433 | 1.475686 | 3.27E-06 | 1.15E-05 |
| CCNE1 | 9.028711 | 25.15364 | 1.478175 | 4.71E-31 | 6.78E-28 |
| FGF12 | 0.24692 | 0.688857 | 1.480163 | 5.21E-19 | 2.77E-17 |
| FAM218A | 0.235676 | 0.663075 | 1.492365 | 4.85E-20 | 3.27E-18 |
| DSCR8 | 0.805807 | 2.269387 | 1.493796 | 1.21E-09 | 8.22E-09 |
| NMU | 4.97651 | 14.05066 | 1.497432 | 1.74E-18 | 7.85E-17 |
| TSHR | 0.146085 | 0.41396 | 1.502686 | 3.99E-08 | 2.02E-07 |
| ELOVL3 | 0.658853 | 1.867218 | 1.502861 | 2.60E-17 | 8.38E-16 |
| CALHM3 | 0.252878 | 0.717511 | 1.504557 | 5.89E-07 | 2.39E-06 |
| ALOX12 | 0.415251 | 1.178311 | 1.504664 | 0.043991 | 0.063752 |
| CNR1 | 0.12332 | 0.350216 | 1.50584 | 1.32E-06 | 5.02E-06 |
| PDE6B | 0.799079 | 2.27053 | 1.506619 | 3.18E-21 | 2.87E-19 |
| MAL | 26.01484 | 73.9566 | 1.507344 | 7.47E-14 | 1.21E-12 |
| SYT13 | 1.192061 | 3.389762 | 1.507725 | 3.73E-26 | 1.30E-23 |
| TNNT1 | 9.171416 | 26.22144 | 1.515531 | 2.25E-24 | 4.65E-22 |
| LHFPL3 | 0.1482 | 0.424489 | 1.518184 | 2.53E-05 | 7.45E-05 |
| ELFN2 | 0.120159 | 0.34496 | 1.521488 | 2.13E-08 | 1.14E-07 |
| RSPO3 | 1.555182 | 4.481224 | 1.526809 | 2.94E-06 | 1.04E-05 |
| CLDN9 | 3.360878 | 9.689217 | 1.527542 | 4.11E-19 | 2.24E-17 |
| NEFH | 1.677095 | 4.838984 | 1.52874 | 0.003233 | 0.006138 |
| PCSK1N | 12.01096 | 34.72856 | 1.531771 | 1.78E-10 | 1.44E-09 |
| DUSP15 | 0.603513 | 1.748269 | 1.534472 | 6.79E-15 | 1.35E-13 |
| NTF4 | 0.165654 | 0.480225 | 1.53554 | 4.55E-14 | 7.72E-13 |
| HENMT1 | 2.142887 | 6.233838 | 1.540565 | 3.19E-27 | 1.36E-24 |
| NLRP7 | 0.251171 | 0.732876 | 1.544899 | 1.93E-08 | 1.04E-07 |
| MX2 | 1.226553 | 3.578915 | 1.544912 | 6.54E-24 | 1.22E-21 |
| C19orf84 | 0.283045 | 0.826341 | 1.545708 | 7.19E-16 | 1.76E-14 |
| PTPRT | 0.152147 | 0.446885 | 1.554441 | 0.000344 | 0.000807 |
| SIX3 | 0.915613 | 2.702464 | 1.561466 | 6.36E-07 | 2.56E-06 |
| GABRE | 0.524637 | 1.551341 | 1.564124 | 1.58E-12 | 1.94E-11 |
| AL589743.1 | 0.144879 | 0.42847 | 1.564341 | 2.95E-17 | 9.45E-16 |
| IDO2 | 0.212712 | 0.629742 | 1.565858 | 0.014336 | 0.023316 |
| FCHO1 | 1.53608 | 4.553176 | 1.56762 | 2.50E-25 | 6.56E-23 |
| CUZD1 | 0.139712 | 0.414184 | 1.567819 | 4.34E-18 | 1.75E-16 |
| GFAP | 0.397901 | 1.18619 | 1.575853 | 2.29E-05 | 6.79E-05 |
| NPW | 2.535109 | 7.566491 | 1.577576 | 2.45E-09 | 1.58E-08 |
| NYAP1 | 0.260963 | 0.778982 | 1.577745 | 7.13E-13 | 9.44E-12 |
| C11orf85 | 0.145898 | 0.436499 | 1.581015 | 1.32E-15 | 3.01E-14 |
| SNCB | 0.170367 | 0.509958 | 1.581735 | 0.000262 | 0.000629 |
| MYLPF | 1.069404 | 3.202105 | 1.582213 | 1.70E-09 | 1.12E-08 |
| HOXD1 | 0.378611 | 1.135443 | 1.584466 | 0.001553 | 0.003166 |
| IGF2BP3 | 0.619916 | 1.859249 | 1.584576 | 1.12E-10 | 9.42E-10 |
| TSPYL5 | 1.360352 | 4.08442 | 1.586152 | 0.035997 | 0.05344 |
| PLXNA4 | 0.730861 | 2.198457 | 1.588822 | 2.61E-05 | 7.67E-05 |
| MSLNL | 0.332666 | 1.005849 | 1.596266 | 7.91E-14 | 1.27E-12 |
| SYT14 | 0.160644 | 0.485788 | 1.596457 | 2.71E-07 | 1.18E-06 |
| NKAIN4 | 1.901124 | 5.75246 | 1.597326 | 7.40E-11 | 6.44E-10 |
| COX6A2 | 0.204901 | 0.621062 | 1.599808 | 2.51E-13 | 3.65E-12 |
| RTBDN | 0.133138 | 0.403965 | 1.601312 | 5.09E-12 | 5.60E-11 |
| MT1H | 3.597083 | 10.91888 | 1.601926 | 2.92E-10 | 2.25E-09 |
| NR0B1 | 0.190813 | 0.579272 | 1.602081 | 2.30E-08 | 1.23E-07 |
| MYT1 | 0.112683 | 0.343696 | 1.60886 | 1.46E-15 | 3.30E-14 |
| DAB1 | 0.132577 | 0.405792 | 1.613907 | 0.00126 | 0.002614 |
| FBXO27 | 1.492069 | 4.591584 | 1.621678 | 3.76E-22 | 4.38E-20 |
| GLDC | 3.033075 | 9.335852 | 1.622 | 2.84E-18 | 1.19E-16 |
| FAM24B | 0.309704 | 0.953852 | 1.622877 | 1.61E-23 | 2.71E-21 |
| IFIT1 | 6.227155 | 19.30856 | 1.632596 | 3.70E-19 | 2.06E-17 |
| GDPD2 | 0.117352 | 0.366167 | 1.641663 | 5.17E-16 | 1.31E-14 |
| ZIK1 | 0.259267 | 0.809923 | 1.643346 | 0.003571 | 0.006713 |
| ERBB2 | 23.34402 | 73.16049 | 1.648012 | 2.62E-11 | 2.50E-10 |
| PTGER1 | 0.550388 | 1.728998 | 1.651415 | 1.81E-07 | 8.14E-07 |
| EGFL6 | 3.62107 | 11.38949 | 1.653216 | 1.61E-16 | 4.56E-15 |
| OR2B6 | 0.325089 | 1.024677 | 1.656263 | 8.09E-15 | 1.58E-13 |
| HPDL | 1.025536 | 3.235272 | 1.657509 | 1.83E-25 | 5.08E-23 |
| FBN3 | 0.952774 | 3.041005 | 1.674343 | 9.93E-14 | 1.57E-12 |
| KCNJ16 | 0.444124 | 1.417958 | 1.674782 | 0.000843 | 0.001823 |
| SSTR1 | 0.329048 | 1.054275 | 1.679884 | 0.000167 | 0.000417 |
| TRO | 1.44198 | 4.627156 | 1.682074 | 1.04E-15 | 2.45E-14 |
| ZNF257 | 0.214881 | 0.690894 | 1.684929 | 1.22E-12 | 1.54E-11 |
| UCHL1 | 18.15096 | 58.39105 | 1.685702 | 5.72E-23 | 7.96E-21 |
| CDH6 | 2.210918 | 7.144882 | 1.692265 | 8.88E-16 | 2.14E-14 |
| GPR158 | 0.161995 | 0.524731 | 1.69563 | 1.55E-21 | 1.49E-19 |
| PNOC | 2.13748 | 6.926436 | 1.696202 | 2.45E-15 | 5.30E-14 |
| JPH3 | 0.219705 | 0.715715 | 1.703819 | 0.003214 | 0.006106 |
| MKRN3 | 0.12222 | 0.399643 | 1.709227 | 1.42E-11 | 1.43E-10 |
| AP3B2 | 0.169672 | 0.555162 | 1.71016 | 5.01E-20 | 3.34E-18 |
| HERC5 | 1.564839 | 5.14082 | 1.715984 | 5.28E-29 | 3.99E-26 |
| CLCNKB | 0.261996 | 0.861346 | 1.717049 | 5.12E-21 | 4.31E-19 |
| TREML2 | 0.144476 | 0.476672 | 1.722167 | 1.70E-09 | 1.12E-08 |
| YBX2 | 1.24349 | 4.113447 | 1.725953 | 2.17E-25 | 5.79E-23 |
| BCAM | 68.36848 | 226.8049 | 1.730048 | 2.19E-18 | 9.61E-17 |
| TUBB4A | 1.5225 | 5.051318 | 1.730218 | 4.15E-19 | 2.25E-17 |
| VGLL1 | 1.777938 | 5.909404 | 1.732808 | 1.45E-13 | 2.21E-12 |
| SLC6A12 | 1.073908 | 3.584981 | 1.739094 | 5.00E-18 | 1.98E-16 |
| ENHO | 0.989062 | 3.306237 | 1.741057 | 8.50E-23 | 1.15E-20 |
| TGM1 | 0.756494 | 2.529329 | 1.741354 | 2.35E-06 | 8.50E-06 |
| DMRT2 | 0.140839 | 0.4746 | 1.752665 | 4.64E-05 | 0.00013 |
| ATP6V1B1 | 6.167402 | 20.83681 | 1.756399 | 2.35E-11 | 2.25E-10 |
| ALK | 0.110589 | 0.37365 | 1.756486 | 1.76E-15 | 3.89E-14 |
| MEOX1 | 1.122047 | 3.796786 | 1.758646 | 5.86E-09 | 3.50E-08 |
| PRSS1 | 0.859951 | 2.930429 | 1.768785 | 1.13E-12 | 1.43E-11 |
| IMPG2 | 0.121676 | 0.414926 | 1.769808 | 0.000273 | 0.000654 |
| PMEL | 1.106996 | 3.779501 | 1.771545 | 0.014321 | 0.023294 |
| LRCH2 | 0.318917 | 1.093437 | 1.777617 | 1.55E-08 | 8.58E-08 |
| BHMT2 | 0.657806 | 2.258318 | 1.779514 | 1.98E-11 | 1.93E-10 |
| FXYD2 | 0.242473 | 0.838103 | 1.789306 | 4.22E-09 | 2.61E-08 |
| IGLON5 | 0.698918 | 2.41687 | 1.789945 | 0.009185 | 0.015676 |
| MYO7B | 0.388763 | 1.346157 | 1.791885 | 2.15E-08 | 1.15E-07 |
| GABRQ | 0.133392 | 0.463851 | 1.797996 | 2.78E-11 | 2.64E-10 |
| SLC22A16 | 0.419755 | 1.46252 | 1.800838 | 3.03E-10 | 2.33E-09 |
| RYR1 | 0.324672 | 1.137135 | 1.80835 | 1.16E-25 | 3.36E-23 |
| DOCK3 | 0.160921 | 0.564478 | 1.810565 | 1.58E-18 | 7.21E-17 |
| SNCG | 6.057797 | 21.31813 | 1.815216 | 5.32E-09 | 3.21E-08 |
| CLDN19 | 0.217976 | 0.768201 | 1.817313 | 1.05E-13 | 1.66E-12 |
| GCGR | 0.381095 | 1.351513 | 1.826354 | 2.11E-13 | 3.10E-12 |
| PRSS50 | 1.115115 | 3.968482 | 1.831394 | 3.52E-13 | 4.98E-12 |
| RNF182 | 0.588653 | 2.097154 | 1.832944 | 1.04E-09 | 7.10E-09 |
| MS4A15 | 0.194965 | 0.695322 | 1.834465 | 2.71E-11 | 2.58E-10 |
| TMEM63C | 0.302869 | 1.080323 | 1.834699 | 5.49E-34 | 2.50E-30 |
| ANKLE1 | 0.617592 | 2.214796 | 1.842448 | 7.62E-21 | 6.03E-19 |
| ZFHX4 | 0.131411 | 0.473675 | 1.849809 | 5.10E-08 | 2.54E-07 |
| GRIK5 | 1.012674 | 3.655006 | 1.851704 | 8.49E-15 | 1.66E-13 |
| KLK6 | 7.387341 | 26.68692 | 1.853006 | 1.57E-10 | 1.28E-09 |
| HIST1H4C | 1.672281 | 6.054484 | 1.856187 | 5.58E-06 | 1.87E-05 |
| DCAF12L1 | 0.494241 | 1.792804 | 1.85893 | 2.03E-11 | 1.98E-10 |
| SMC1B | 0.174711 | 0.635762 | 1.863513 | 1.47E-23 | 2.58E-21 |
| KCNK9 | 0.146278 | 0.533736 | 1.867412 | 9.84E-20 | 6.27E-18 |
| ZDBF2 | 0.183843 | 0.674079 | 1.874445 | 6.37E-07 | 2.56E-06 |
| NUPR1L | 1.510475 | 5.555856 | 1.879007 | 1.68E-17 | 5.69E-16 |
| MAG | 0.228131 | 0.845805 | 1.890463 | 4.65E-12 | 5.16E-11 |
| ANKRD33B | 0.291922 | 1.083301 | 1.891778 | 8.34E-21 | 6.53E-19 |
| KCNQ2 | 0.25522 | 0.947693 | 1.892678 | 0.000119 | 0.000305 |
| ZNF311 | 0.217958 | 0.812587 | 1.898472 | 1.35E-17 | 4.72E-16 |
| PLAG1 | 0.297126 | 1.122183 | 1.917163 | 2.98E-24 | 5.98E-22 |
| KLRG2 | 0.429338 | 1.622163 | 1.917732 | 2.46E-28 | 1.46E-25 |
| TDRD12 | 0.284685 | 1.078072 | 1.921015 | 2.77E-10 | 2.15E-09 |
| DMRT1 | 0.277296 | 1.054032 | 1.92642 | 7.40E-13 | 9.75E-12 |
| GRB7 | 10.28568 | 39.23044 | 1.931336 | 6.11E-16 | 1.52E-14 |
| BMP3 | 0.596837 | 2.277417 | 1.931989 | 0.010335 | 0.017431 |
| TCEAL5 | 0.676586 | 2.587145 | 1.935016 | 1.28E-10 | 1.06E-09 |
| CNTFR | 1.406059 | 5.377677 | 1.935326 | 1.93E-10 | 1.55E-09 |
| CYS1 | 2.179135 | 8.473054 | 1.959127 | 0.004349 | 0.008003 |
| PPP2R2B | 0.127029 | 0.495261 | 1.963028 | 6.05E-09 | 3.61E-08 |
| WNT7A | 7.050715 | 27.51683 | 1.964473 | 3.16E-14 | 5.48E-13 |
| FBXO17 | 1.187679 | 4.654824 | 1.970581 | 7.36E-19 | 3.71E-17 |
| LMO3 | 0.697035 | 2.739662 | 1.974695 | 1.30E-14 | 2.47E-13 |
| PTX3 | 0.62656 | 2.46828 | 1.977982 | 1.69E-18 | 7.67E-17 |
| FXYD7 | 0.1485 | 0.587698 | 1.984614 | 1.99E-08 | 1.08E-07 |
| SPOCK3 | 0.210531 | 0.834917 | 1.987603 | 2.60E-06 | 9.33E-06 |
| CORO6 | 0.374774 | 1.494666 | 1.995732 | 7.16E-10 | 5.08E-09 |
| FOXI3 | 0.171289 | 0.687059 | 2.004004 | 9.00E-10 | 6.25E-09 |
| PPARGC1A | 0.165759 | 0.672602 | 2.020664 | 7.39E-13 | 9.75E-12 |
| COL22A1 | 0.34049 | 1.400277 | 2.040027 | 5.36E-08 | 2.66E-07 |
| DLL3 | 0.498889 | 2.059363 | 2.045408 | 2.66E-16 | 7.30E-15 |
| HOXA4 | 0.571481 | 2.370814 | 2.052605 | 5.82E-13 | 7.86E-12 |
| ZNF334 | 0.207466 | 0.863258 | 2.056918 | 1.89E-12 | 2.27E-11 |
| CLDN16 | 0.99601 | 4.209224 | 2.079322 | 8.76E-14 | 1.40E-12 |
| MAFA | 0.187809 | 0.796664 | 2.084709 | 1.05E-10 | 8.90E-10 |
| PLAC1 | 0.155206 | 0.659088 | 2.086287 | 1.45E-11 | 1.46E-10 |
| ADRA1B | 0.503534 | 2.153051 | 2.096223 | 8.50E-17 | 2.56E-15 |
| RSPO4 | 0.744236 | 3.184175 | 2.097088 | 4.37E-21 | 3.74E-19 |
| NPR1 | 2.802533 | 12.13223 | 2.114042 | 6.37E-18 | 2.42E-16 |
| PNMA3 | 1.172297 | 5.076999 | 2.114637 | 1.25E-22 | 1.61E-20 |
| KLK8 | 1.850779 | 8.034023 | 2.11799 | 6.17E-12 | 6.72E-11 |
| ANKRD2 | 0.27118 | 1.180231 | 2.121746 | 5.64E-12 | 6.17E-11 |
| DOK5 | 0.622847 | 2.733258 | 2.133672 | 3.04E-12 | 3.51E-11 |
| CYP2W1 | 0.936221 | 4.13191 | 2.141889 | 0.01534 | 0.02478 |
| FOXD3 | 0.091467 | 0.407559 | 2.155683 | 8.80E-09 | 5.08E-08 |
| CRB2 | 0.275735 | 1.230146 | 2.157475 | 5.31E-05 | 0.000147 |
| GPRIN2 | 0.523615 | 2.34271 | 2.161599 | 9.33E-23 | 1.22E-20 |
| ZDHHC19 | 0.13734 | 0.616119 | 2.165456 | 1.11E-21 | 1.12E-19 |
| FLG | 0.102603 | 0.470745 | 2.197871 | 0.000512 | 0.001157 |
| SLITRK2 | 0.125688 | 0.580889 | 2.208415 | 4.90E-07 | 2.02E-06 |
| ZNF229 | 0.131231 | 0.616667 | 2.232385 | 4.86E-10 | 3.56E-09 |
| COMP | 4.61992 | 22.10566 | 2.258476 | 5.96E-05 | 0.000163 |
| CRTAC1 | 0.759307 | 3.648486 | 2.264543 | 1.65E-14 | 3.06E-13 |
| RNF212 | 0.491578 | 2.370717 | 2.269831 | 2.01E-14 | 3.65E-13 |
| SLC14A2 | 0.205634 | 0.996087 | 2.276195 | 0.010785 | 0.018116 |
| EEF1A2 | 8.057591 | 39.22454 | 2.283336 | 1.16E-05 | 3.64E-05 |
| CDKN2A | 5.77412 | 28.23079 | 2.289596 | 1.14E-31 | 2.60E-28 |
| SOX11 | 0.241516 | 1.181524 | 2.290456 | 2.29E-08 | 1.22E-07 |
| DPYSL5 | 0.163335 | 0.801347 | 2.294596 | 1.48E-08 | 8.22E-08 |
| PQLC2L | 0.226471 | 1.114149 | 2.298544 | 1.36E-12 | 1.69E-11 |
| AOC1 | 15.23037 | 75.04052 | 2.300719 | 4.02E-10 | 3.00E-09 |
| WT1 | 1.553291 | 7.721612 | 2.313574 | 3.34E-08 | 1.73E-07 |
| COL4A4 | 0.213926 | 1.073217 | 2.326761 | 1.18E-11 | 1.22E-10 |
| SGCG | 0.126133 | 0.635057 | 2.331936 | 4.56E-09 | 2.80E-08 |
| EYA4 | 0.129946 | 0.659083 | 2.342543 | 1.67E-19 | 1.03E-17 |
| MAGEA4 | 0.871229 | 4.444253 | 2.350817 | 3.17E-12 | 3.64E-11 |
| CDH18 | 0.241486 | 1.237057 | 2.356899 | 9.44E-19 | 4.56E-17 |
| IGF2 | 71.06147 | 368.0524 | 2.372772 | 0.010647 | 0.017896 |
| SLC6A13 | 0.155817 | 0.807473 | 2.37356 | 2.27E-16 | 6.33E-15 |
| GAL3ST3 | 0.564102 | 2.975654 | 2.39918 | 1.96E-14 | 3.58E-13 |
| LBP | 2.75535 | 14.61569 | 2.40721 | 0.029415 | 0.044508 |
| SLC6A11 | 0.080109 | 0.426127 | 2.411256 | 1.97E-27 | 9.23E-25 |
| MYOD1 | 0.104162 | 0.554645 | 2.412728 | 1.38E-12 | 1.72E-11 |
| SLC6A15 | 0.092865 | 0.495835 | 2.416655 | 2.59E-14 | 4.56E-13 |
| HIF3A | 0.540669 | 2.915345 | 2.43085 | 5.82E-24 | 1.12E-21 |
| DLGAP3 | 0.379922 | 2.074607 | 2.449065 | 1.34E-24 | 2.94E-22 |
| SLC7A10 | 0.115704 | 0.638372 | 2.463958 | 4.62E-20 | 3.16E-18 |
| TMPRSS6 | 0.155333 | 0.863668 | 2.475112 | 3.07E-16 | 8.22E-15 |
| UPK3B | 1.712524 | 9.527406 | 2.47596 | 7.61E-14 | 1.23E-12 |
| KCNK2 | 0.128646 | 0.727969 | 2.500476 | 1.70E-10 | 1.38E-09 |
| NKAIN2 | 0.072152 | 0.419087 | 2.538135 | 0.000418 | 0.000964 |
| MAGEA11 | 0.364165 | 2.134374 | 2.55115 | 3.56E-08 | 1.83E-07 |
| TRHDE | 0.113883 | 0.674552 | 2.566377 | 6.33E-11 | 5.56E-10 |
| RHOXF1 | 0.397531 | 2.378858 | 2.581128 | 0.00367 | 0.00687 |
| COL4A3 | 0.088593 | 0.536497 | 2.598309 | 2.78E-09 | 1.77E-08 |
| HTR3A | 0.666846 | 4.061341 | 2.60653 | 7.22E-09 | 4.23E-08 |
| PCDH10 | 0.185265 | 1.143393 | 2.625659 | 8.26E-10 | 5.78E-09 |
| KLK7 | 2.329888 | 14.71407 | 2.658864 | 4.75E-13 | 6.56E-12 |
| MAGEA10 | 0.180751 | 1.146958 | 2.665734 | 8.39E-08 | 4.03E-07 |
| CLDN6 | 8.370724 | 53.5733 | 2.67809 | 8.54E-19 | 4.19E-17 |
| COX8C | 0.154012 | 1.027391 | 2.737868 | 0.035056 | 0.052202 |
| PVALB | 0.20486 | 1.407709 | 2.780643 | 5.66E-13 | 7.69E-12 |
| CTCFL | 0.270643 | 1.897252 | 2.809447 | 5.85E-18 | 2.25E-16 |
| MCCD1 | 0.253505 | 1.780755 | 2.812405 | 4.84E-11 | 4.35E-10 |
| PAGE2B | 0.329852 | 2.38893 | 2.856476 | 2.68E-08 | 1.41E-07 |
| SST | 20.46919 | 148.7361 | 2.861229 | 2.61E-19 | 1.52E-17 |
| CALCA | 2.579988 | 19.05042 | 2.884386 | 1.38E-07 | 6.34E-07 |
| AARD | 0.221244 | 1.694053 | 2.936768 | 3.65E-25 | 9.39E-23 |
| KLK5 | 1.338838 | 10.60106 | 2.985155 | 1.37E-09 | 9.21E-09 |
| PDCL2 | 0.235864 | 1.875221 | 2.991036 | 1.77E-23 | 2.91E-21 |
| VSTM2B | 0.07324 | 0.601283 | 3.037348 | 3.87E-12 | 4.36E-11 |
| L1CAM | 1.626962 | 13.5194 | 3.054779 | 2.07E-28 | 1.28E-25 |
| UPK2 | 1.203997 | 10.2605 | 3.091198 | 4.92E-06 | 1.66E-05 |
| COL9A1 | 0.88797 | 8.100413 | 3.189412 | 1.11E-15 | 2.58E-14 |
| NPY | 0.296784 | 2.740802 | 3.207111 | 1.42E-09 | 9.56E-09 |
| CALCB | 0.550722 | 5.742779 | 3.382352 | 2.12E-08 | 1.14E-07 |
| FOLR3 | 0.145402 | 1.690496 | 3.539324 | 4.14E-18 | 1.67E-16 |
| APOA1 | 2.714122 | 31.57487 | 3.540219 | 3.88E-06 | 1.34E-05 |
| TNMD | 0.059859 | 0.89726 | 3.905888 | 0.001072 | 0.002262 |
| PAGE5 | 0.067635 | 1.142181 | 4.077875 | 0.012584 | 0.020748 |
| XAGE2B | 0.169698 | 3.240815 | 4.255311 | 2.51E-20 | 1.78E-18 |
| ACTL8 | 0.266655 | 5.769838 | 4.435487 | 3.98E-21 | 3.45E-19 |
| PAGE2 | 0.274227 | 7.550596 | 4.783151 | 3.21E-09 | 2.02E-08 |
| TKTL1 | 0.206116 | 5.808235 | 4.816572 | 3.30E-10 | 2.52E-09 |

**Supplementary Table 2** A total of 1058 differentially expressed genes (DEGs), including 525 upregulated genes and 533 downregulated genes, were identified based on p < 0.05 and log2∣FC∣> 1 screening standard.
