# Supplementary material for: Significance of TP53 Mutational Status-Associated Signature in the Progression and Prognosis of Endometrial Carcinoma
Source: Oxid Med Cell Longev. 2022 Jul 6;2022:1817339. doi: 10.1155/2022/1817339 (PMC9280614; doi:10.1155/2022/1817339)
Supplement: Supplementary 3 — Supplementary Table 3: univariate Cox regression preliminarily screened 50 DEGs with the prognosis value. [file 1817339.f3.docx]

| id | HR | HR.95L | HR.95H | pvalue |
| --- | --- | --- | --- | --- |
| STAC | 2.414711 | 1.837331 | 3.173531 | 2.56E-10 |
| KCNK9 | 1.439595 | 1.25486 | 1.651526 | 1.99E-07 |
| WNT10A | 1.042624 | 1.02544 | 1.060097 | 8.54E-07 |
| DOK5 | 1.061961 | 1.036147 | 1.088419 | 1.68E-06 |
| C19orf33 | 1.001501 | 1.00086 | 1.002143 | 4.49E-06 |
| GLOD5 | 1.125339 | 1.069504 | 1.18409 | 5.42E-06 |
| RSPO4 | 1.083292 | 1.045476 | 1.122476 | 1.02E-05 |
| ADAMTS16 | 1.114497 | 1.061913 | 1.169684 | 1.10E-05 |
| RBP2 | 1.0912 | 1.048391 | 1.135757 | 1.92E-05 |
| PAGE5 | 1.033356 | 1.017869 | 1.04908 | 2.06E-05 |
| ZNF829 | 1.68096 | 1.319717 | 2.141085 | 2.58E-05 |
| MYT1 | 1.767427 | 1.353195 | 2.308462 | 2.92E-05 |
| OR2W3 | 1.365016 | 1.17888 | 1.580542 | 3.18E-05 |
| GDPD2 | 1.442746 | 1.21208 | 1.717309 | 3.73E-05 |
| NMU | 1.022078 | 1.011484 | 1.032783 | 3.99E-05 |
| MAL | 1.002545 | 1.001315 | 1.003777 | 4.97E-05 |
| HIF3A | 1.051987 | 1.026458 | 1.078151 | 5.27E-05 |
| PTPRT | 1.190596 | 1.093541 | 1.296265 | 5.79E-05 |
| SLC8A1 | 1.317766 | 1.148328 | 1.512205 | 8.51E-05 |
| EPHB2 | 1.077015 | 1.037374 | 1.118171 | 0.000105 |
| CLCN4 | 1.290199 | 1.133923 | 1.468011 | 0.00011 |
| SLC38A1 | 1.021183 | 1.010292 | 1.032192 | 0.000127 |
| ERBB2 | 1.001355 | 1.00066 | 1.002051 | 0.000132 |
| ATP6V1B1 | 1.009345 | 1.004527 | 1.014186 | 0.000139 |
| LRRN1 | 1.026223 | 1.01257 | 1.040061 | 0.000152 |
| KCNK6 | 0.90359 | 0.857295 | 0.952384 | 0.000158 |
| SLC6A11 | 1.625992 | 1.261411 | 2.095945 | 0.000175 |
| GRB7 | 1.003118 | 1.001455 | 1.004785 | 0.000236 |
| MUCL1 | 1.002248 | 1.001047 | 1.00345 | 0.000242 |
| TMEM63C | 1.293078 | 1.126682 | 1.484047 | 0.000255 |
| HPDL | 1.097126 | 1.043016 | 1.154043 | 0.000328 |
| LGALS7 | 1.203416 | 1.08777 | 1.331357 | 0.000328 |
| MX2 | 1.125168 | 1.055015 | 1.199985 | 0.00033 |
| TPX2 | 1.016737 | 1.00748 | 1.026078 | 0.000375 |
| KLHL30 | 1.300941 | 1.125045 | 1.504338 | 0.000386 |
| GFAP | 1.022826 | 1.010159 | 1.035652 | 0.000386 |
| L1CAM | 1.014495 | 1.006457 | 1.022598 | 0.000391 |
| KCNJ12 | 1.10407 | 1.045264 | 1.166185 | 0.000392 |
| DCAF12L1 | 1.145853 | 1.062313 | 1.235963 | 0.000423 |
| TMTC1 | 1.072933 | 1.031727 | 1.115785 | 0.000426 |
| TTK | 1.103433 | 1.044352 | 1.165857 | 0.000456 |
| ZDHHC1 | 0.922227 | 0.880205 | 0.966254 | 0.000667 |
| FAM131C | 1.239696 | 1.095031 | 1.403474 | 0.000689 |
| RAB39B | 1.485716 | 1.181609 | 1.86809 | 0.000704 |
| FAM24B | 1.433982 | 1.164005 | 1.766578 | 0.000707 |
| SLITRK2 | 1.169434 | 1.067145 | 1.281528 | 0.000804 |
| PGR | 0.95544 | 0.930221 | 0.981343 | 0.000838 |
| UPK3B | 1.010659 | 1.004387 | 1.01697 | 0.000844 |
| MSX1 | 0.998149 | 0.997057 | 0.999242 | 0.00091 |
| GLIS2 | 1.02781 | 1.01116 | 1.044734 | 0.000995 |

**Supplementary Table 3** Univariate Cox regression preliminarily screened 50 DEGs with the prognosis value.
